# Dolutegravir plus lamivudine downregulates cellular stress responses vs. three-drug HIV regimens

**DOI:** 10.1097/QAD.0000000000004198

**Published:** 2025-04-01

**Authors:** Victoria Rios-Vazquez, Wilhelm A.J.W. Vos, Marc J.T. Blaauw, Louise E. Van Eekeren, Albert L. Groenendijk, Adriana Navas, Nadira Vadaq, Leo A.B. Joosten, Mihai G. Netea, Willem L. Blok, Janneke E. Stalenhoef, Lambert Van Den Heuvel, Andre J.A.M. Van Der Ven, Jan Van Lunzen

**Affiliations:** a Department of Internal Medicine and Radboud Center of Infectious Diseases; b Department of Genetics and Pediatrics, Radboudumc, Radboud University, Nijmegen; c Department of Internal Medicine and Infectious Diseases, OLVG, Amsterdam; d Department of Internal Medicine and Infectious Diseases, Elizabeth-Tweesteden Ziekenhuis; e Department of Internal Medicine and Department of Medical Microbiology and Infectious Diseases, ErasmusMC, Erasmus University, Rotterdam, The Netherlands; f Department of Medical Genetics, Iuliu Hatieganu University of Medicine and Pharmacy, Cluj-Napoca, Romania; g Department of Immunology and Metabolism, Life and Medical Sciences Institute, University of Bonn, Bonn, Germany.

**Keywords:** antiretroviral therapy, dolutegravir, HIV, multiomics, reverse transcriptase inhibitors

## Abstract

**Objective:**

To compare the systemic and immune effects of two-drug regimens (2DR) and three-drug regimens (3DR) in people with HIV (PWH).

**Design:**

In a cross-sectional study, multiomics data were analyzed in dolutegravir (DTG) plus lamivudine (3TC) 2DR and 3DR comprising DTG with two nucleoside reverse transcriptase inhibitors (NRTIs).

**Methods:**

Data from the 2000HIV cohort of virally suppressed PWH on combination antiretroviral treatment (cART) regimens were analyzed. Groups included DTG + 3TC (*n* = 191), DTG + 3TC + abacavir (ABC) (*n* = 188), and DTG + tenofovir disoproxil fumarate or tenofovir alafenamide (TDF/TAF) + emtricitabine (FTC) (*n* = 115). Systemic functions were assessed via plasma protein profiling (Olink Explore, 2367 proteins), while peripheral blood mononuclear cells (PBMCs) were used to evaluate immune effects by analyzing Bulk RNA-Seq and ex-vivo cytokine production capacity.

**Results:**

Plasma protein analysis revealed that 2DR associated to lower protein expression and pathways related to metabolism, stress responses, chemokine signaling, and immune responses compared to 3DR. Four proteins – CXCL8, DDC, PMM2, and EPS8L2 – were consistently downregulated in 2DR. Differential gene expression analysis identified 17 overlapping downregulated genes across all 3DR vs. 2DR comparisons, linked to chromatin structure, cellular senescence, stress response, and cytokine activity. Cytokine production was similar across 2DR and 3DR groups, except for enhanced interleukin (IL)-17 production in DTG + TDF + FTC users.

**Conclusions:**

Reducing NRTIs in DTG-based 2DR, particularly by omitting ABC or TAF/TDF, suggests decreased activation of stress response and immune-related pathways. Importantly, the functional capacity of circulating immune cells remains largely unchanged between 2DR and 3DR.

## Introduction

Combination antiretroviral treatment (cART) significantly reduces HIV-related morbidity and mortality, improves quality of life, and prevents transmission by restoring immune function, reducing inflammation, and suppressing HIV replication, while trying to minimize therapy side effects [1]. Current guidelines recommend a three-drug regimen (3DR) comprising two nucleoside reverse transcriptase inhibitors (NRTIs) and an integrase strand transfer inhibitor (INSTI), such as dolutegravir (DTG), bictegravir, or cabotegravir [2]. WHO's first-line recommendation includes dolutegravir with tenofovir disoproxil fumarate and lamivudine (3TC) or emtricitabine (FTC) [3].

Recent efforts have explored reducing cART regimens to minimize toxicity, despite concerns over potential increases in immune activation from reduced HIV suppression [4]. In PWH without chronic hepatitis B and a viral load < 500 000 copies/ml, the two-drug regimen (2DR) of dolutegravir and lamivudine (DTG+3TC) has proven safe and effective [5,6]. DTG + 3TC is noninferior to 3DR in terms of safety, tolerability, and efficacy, while avoiding tenofovir-related side effects like renal toxicity and bone loss [7,8]. Although newer NRTIs like TDF and tenofovir alafenamide (TAF) exhibit reduced toxicity compared to first-generation NRTIs, concerns persist regarding their contribution to systemic inflammation and immune cell function impairment via oxidative stress and mitochondrial damage [9–15]. These effects may underlie ART-associated comorbidities, including cardiovascular disease and metabolic syndromes [16,17].

High-throughput *omics* technologies, such as plasma proteomics and immune cell transcriptomics, enable detailed profiling of systemic and immune responses [16,17]. To assess systemic inflammation, plasma proteomics should be accompanied by functional assessment of immune cells, such as cytokine responses ex vivo [18]. Currently, DTG+3TC is the most recommended 2DR for initial cART in ART-naïve adults across most guidelines [6]. We hypothesize dolutegravir-based 2DR (DTG + 3TC) and 3DR (DTG + 2NRTIs) differentially impacts immune responses and systemic profiles, particularly in reducing NRTI-induced toxicities. The aim of this study is to compare the immune responses and systemic effects of 2DR and 3DR by analyzing plasma proteome, circulating immune cell transcriptome, and ex-vivo cytokine responses of peripheral blood mononuclear cells (PBMCs).

## Methods

### Study population

**Fig. 1:**
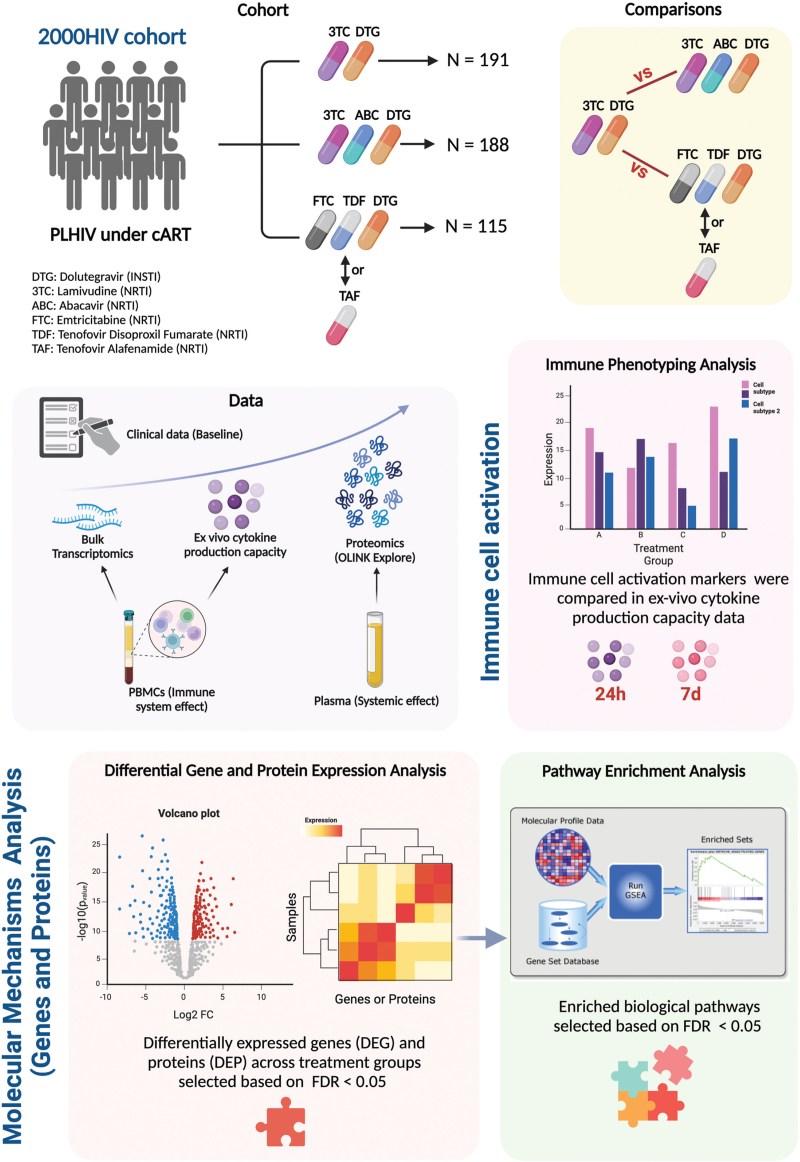
Overview of study design and multiomics analysis in the 2000HIV cohort.

494 participants were selected from the 2000HIV study (NTC03994835) [19]. The selection was based on receiving a DTG-based regimen. Participants were divided into treatment groups: 2DR (DTG + 3TC, *n* = 191) and 3DR, which included DTG + 3TC + ABC (*n* = 188) and DTG + TDF/TAF + FTC (*n* = 115), further subdivided into DTG + TDF + FTC (*n* = 62) and DTG + TAF + FTC (*n* = 53). Eligibility criteria, detailed in previous publications [19], included age ≥18 years, HIV-1 positivity, cART for ≥6 months, and viral load <200 copies/ml (Fig. 1). Exclusion criteria comprised pregnancy, detectable viral hepatitis B/C DNA by PCR, or acute infections.

This study compared the systemic and immune-specific effects of two-drug regimens (2DR) vs. three-drug regimens (3DR) in virally suppressed people with HIV (PWH) under dolutegravir-based combination antiretroviral therapy (cART). The cohort included participants on 2DR (DTG + 3TC, *n* = 191) and three 3DR subgroups: DTG + 3TC + ABC (*n* = 188), DTG + TDF + FTC (*n* = 62), and DTG + TAF + FTC (*n* = 53). Key comparisons are highlighted between 2DR and each 3DR regimen. Data layers included clinical data (baseline), plasma proteomics (systemic effects; 2367 proteins, Olink Explore), transcriptomics (PBMC gene expression; bulk RNA sequencing), and ex vivo cytokine production (immune activation; PBMC stimulation at 24 h and 7 days). Analyses included differential gene and protein expression, immune phenotyping, and pathway enrichment to identify molecular signatures and pathways associated with cART regimens. Immune cell activation markers were evaluated using ex vivo cytokine production, while differentially expressed genes (DEGs) and proteins (DEPs) were identified [false discovery rate (FDR) < 0.05)] and mapped to enriched biological pathways using gene set enrichment analysis (GSEA). Created with Biorender.

### Ethics

The 2000HIV study protocol was approved by the accredited medical research ethics committee Nijmegen (NL68056.091.81). Informed consent was obtained from all participants. The principles of the Declaration of Helsinki were followed.

### High throughput targeted proteomics (Olink Explore)

Plasma proteomics was conducted using the Olink Explore 3072 panel, which employs a proximity extension assay where proteins bind to oligonucleotide-labeled antibodies, as detailed in prior literature [20,21]. These sequences are amplified and quantified by qPCR or NGS, reflecting protein concentrations. For the 2000HIV cohort, 2367 proteins were analyzed after normalization (Log2 scale) and adjustment for proximity extension and plate controls.

### High-throughput transcriptomics (bulk RNA sequencing)

Bulk RNA sequencing of PBMCs was performed for the 2000HIV cohort, following detailed protocols [19,22]. Quality control excluded samples with low mapping reads, biological discrepancies, or contamination, resulting in available data for 1853 samples. Genes with <10 counts in the smallest group were removed, retaining ∼16 000 out of 58 347 for downstream analysis. Quality control ensured robust data for subsequent analyses.

### Ex-vivo cytokine production

PBMCs were isolated and cultured ex vivo to assess cytokine production upon stimulation with a panel of inactivated pathogens. Stimulation was conducted for 24 h with Poly I:C, lipopolysaccharide (LPS), Imiquimod, human recombinant interleukin (IL)-1α, HIV envelope protein (HIV-ENV), cytomegalovirus (CMV), and *S. pneumoniae*, as well as for 7 days with *E. coli*, *S. aureus*, *S. pneumoniae*, *M. tuberculosis*, *C. albicans* (conidia), phytohemagglutinin (PHA), and *C. albicans* (hyphae). Cytokine responses were categorized into two groups based on stimulation duration: a 24-h group including IL-1β, IL-1Ra, IL-6, IL-8, IL-10, MCP-1, MIP1-α, and tumor necrosis factor (TNF), which are mainly monocyte-related cytokines, and a 7-day group comprising IL-5, IL-10, IL-17, IL-22, and IFN-γ, that are mainly related to lymphocyte production. Following stimulation, cell-free supernatants were harvested and stored until analysis. Cytokine concentrations were determined using enzyme-linked immunosorbent assays (ELISA), as previously described [19].

### Statistics

#### Demographic characteristics assessment

Demographic characteristics of the participants were assessed using chi-square test and Kruskal-Wallis test for categorical and numerical variables, respectively (Tables S1, Supplemental Digital Content 1).

#### Treatment groups comparisons

The study compared two-drug regimens (2DR) vs. three-drug regimens (3DR) to assess the impact of different NRTI combinations on the plasma proteome, gene expression, and immune responses in people with HIV (PWH). Comparisons included:

(1) 2DR: DTG + 3TC vs. 3DR: DTG + 3TC + ABC – to examine the effects of adding a second NRTI (ABC).
(2) 2DR: DTG + 3TC vs. 3DR: DTG + TDF/TAF + FTC – to evaluate if changes observed with ABC persist with alternative NRTI backbones.
(3) 2DR: DTG + 3TC vs. 3DR: DTG + TDF + FTC and 3DR: DTG + TAF + FTC – to distinguish individual effects of TDF and TAF in 3DR.

TAF and TDF were grouped for the primary 2DR vs. 3DR comparison (2) to ensure a balanced statistical analysis, but their individual effects were also assessed separately (3).

#### Plasma proteome differential expression

The impact of the antiretroviral regimens on the plasma proteome was analyzed using Olink Explore to identify differentially expressed proteins (DEPs) across treatment groups. Differential expression was calculated using the limma workflow [23] and corrected for multiple testing using the Benjamini–Hochberg (BH) procedure. Potential confounders identified through PCA analysis of proteomics data were age, sex, and sample collection season (Fig. S1, Supplemental Digital Content 1). The aim was to identify molecular signatures that distinguished different treatment groups in the 2000HIV study. Proteins with an adjusted *P*-value < 0.05 and Log_2_ fold change (Log_2_FC) ≠0 were considered differentially expressed.

#### Transcriptomics differential expression

Gene expression was assessed through bulk RNA sequencing of PBMCs, comparing the same 2DR and 3DR groups as in the previous analysis. Differentially expressed genes (DEGs) were identified using the DESeq2 workflow [24], applying negative binomial generalized linear models, BH for multiple testing correction, and the “apeglm” method for Log_2_FC shrinkage. Covariates included in the models were like those in the proteomics analysis, with additional adjustments for sample collection center and plate, specific for this data layer. Genes showing adjusted *P*-values < 0.05 and Log_2_FC >0.58 (fold change > 1.5) were considered differentially expressed.

#### Ex-vivo cytokine production analysis

Ex-vivo cytokine production from PBMCs was measured after stimulation for 24 h or 7 days using various agents [19]. Cytokine production levels were compared between treatment groups, adjusting for covariates such as age, sex, and sample collection season. Rank-based regression modeling was used, and results were corrected for multiple testing using the FDR method. The ’rfit’ function from the ’rms’ package [25] was employed to fit linear models to each cytokine variable. To address the right skewness of the data, a ’Bent1’ transformation was applied. Significant cytokine associations were defined based on an adjusted *P*-value < 0.05 after correcting for confounding factors.

#### Pathway enrichment analysis

Pathway analysis identified significant pathways associated with proteomics and transcriptomics markers. Markers from differential expression analyses were ranked by adjusted p-values, selecting the top 100 markers for each comparison, irrespective of regulation direction. These were split into up- and down-regulated groups for separate analysis. Only measured markers were used as background. Enrichment for proteomics was performed using DAVID (GO terms, Reactome, KEGG, WikiPathways). Transcriptomics enrichment used the clusterProfiler package to identify enriched GO terms, KEGG, and HALLMARK pathways among top significant genes. Pathways with an adjusted *P*-value < 0.05 were considered significant. Top 10 pathways per comparison were visualized as balloon plots using clusterProfiler [26–28].

#### Overrepresentation analysis of common differentially expressed proteins and differentially expressed genes

Overlapping significant DEPs and DEGs (adjusted *P*-value < 0.05) were analyzed using overrepresentation analysis. This focused on downregulated DEPs and DEGs shared between the 2DR vs. 3DR comparisons. The analysis was performed using g:Profiler g:GOSt [29], with the total proteins and genes from differential expression analysis as the reference set. Significant enriched terms were identified by BH-adjusted *P*-values < 0.05.

## Results

### Demographic and clinical characteristics

Demographic and clinical characteristics of the participants are detailed in Tables S1, Supplemental Digital Content 1. Significant differences were observed among the treatment groups in age, HIV duration, COVID vaccination, and the most recent CD4^+^ cell count. These variables were assessed for their potential confounding effects on the *omics* layer analyses and, where necessary, adjustments were made.

### Proteomics reveal differential downregulation of metabolic and immune pathways in 2DR with one NRTI compared to 3DR with Two NRTIs

To investigate the systemic molecular effects of NRTIs in cART, we analyzed plasma proteomics in PWH on DTG-based regimens containing two NRTIs (3DR) or one NRTI (2DR). As a secondary objective, the individual effects of TDF and TAF were also assessed.

**Fig. 2:**
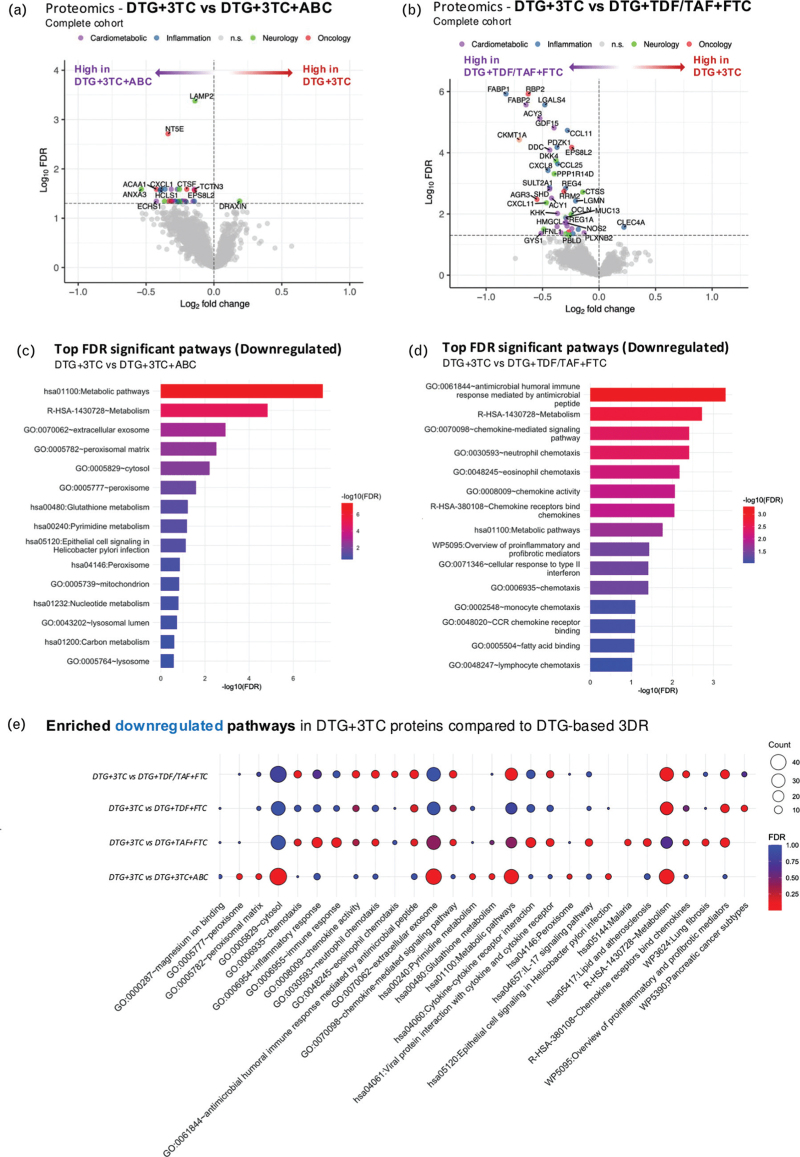
Differential protein expression and pathway enrichment in PWH using 2DR and 3DR.

First, 2DR: DTG + 3TC compared to 3DR: DTG + 3TC + ABC 27 downregulated (log_2_FC: −0.53 to −0.14) and one upregulated (DRAXIN, log_2_FC: 0.19) DEPs (FDR < 0.05) in 2DR (Fig. 2a). Pathway analysis revealed enrichment (FDR < 0.05, lower in 2DR) in metabolic pathways, including ‘metabolic pathways’ (hsa01100) ‘metabolism’ (R-HSA-1430728), and ‘Extracellular exosome’ (GO:0070062), as well as pathways linked to stress response and cellular compartmentalization (e.g., peroxisomal matrix (GO:0005782)). Upregulated pathways were not analyzed due to the presence of only one DEP. Detailed results can be found in Tables S2 and S10, Supplemental Digital Content 1.

(a, b) Volcano plots of the differentially expressed proteins between PWH under DTG + 3TC vs. DTG + 3TC + ABC (a) and DTG + TDF/TAF + FTC (b). X-axis depicts Log_2_ fold change of differentially expressed proteins and Y-axis depicts the −Log_10_(FDR P value). Differential expression analysis was performed using age, sex, and center (RUMC) as covariates. (c, d) Barplot with the top 15 most FDR *P*-value significant enriched pathways by the downregulated proteins in the comparison between DTG + 3TC vs. DTG + 3TC + ABC (a) and DTG + TDF/TAF + FTC (b). X axis depicts the -log_10_(FDR *P*-value) significance of the pathway and the Y-axis the pathway terms. (e) Balloon plot of the top 10 most significant (FDR *P*-value) downregulated pathways per 2DR vs. 3DR comparison. Color indicates FDR *P*-value significance and the size the count of the proteins contributing to the pathway enrichment.

Next, we explored whether similar differences could also be observed with a different NRTI backbone. Comparing 2DR: DTG + 3TC to 3DR: DTG+TDF/TAF + FTC, we observed 42 downregulated (log_2_FC: −0.8 to −0.1) and one upregulated (CLEC4A, log_2_FC: 0.2) DEPs in 2DR (FDR < 0.05) (Fig. 2b). Pathway analysis again highlighted significant enrichment in metabolic pathways (lower in 2DR), including ’Metabolic pathways’ (hsa01100) and ‘metabolism’ (R-HSA-1430728). Additionally, significant downregulation was observed in pathways associated with chemokine signaling and immune responses, such as ‘chemotaxis’ (GO:0006935) and ‘chemokine activity’ (GO:0008009). Furthermore, pathways involved in cellular response to type II interferon (GO:0071346), chemokine receptor binding (R-HSA-380108), chemokine-mediated signaling (GO:0070098), proinflammatory and profibrotic mediators (WP5095) were also downregulated as detailed in Tables S3 and S11, Supplemental Digital Content 1.

The individual effects of TDF and TAF as NRTIs in 3DR were assessed by comparing 2DR: DTG + 3TC with 3DR subgroups (DTG + TDF + FTC and DTG + TAF + FTC). The 2DR: DTG + 3TC compared with 3DR: DTG + TDF + FTC showed 63 downregulated (log_2_FC: −1.4 to −0.1) and two upregulated (Cathepsin E (CTSE) with log_2_FC:0.5, and carboxypeptidase A4 (CPA4) with log_2_FC: 0.2) DEPs in 2DR, while the comparison with 3DR: DTG + TAF + FTC revealed only one downregulated DEP: C–X–C motif chemokine ligand 8 (CXCL8, log_2_FC: −0.6) (Fig. S3B and Tables S4–S5, Supplemental Digital Content 1). Pathway analysis of these comparisons revealed no statistically significant enrichments (FDR < 0.05).

**Fig. 3:**
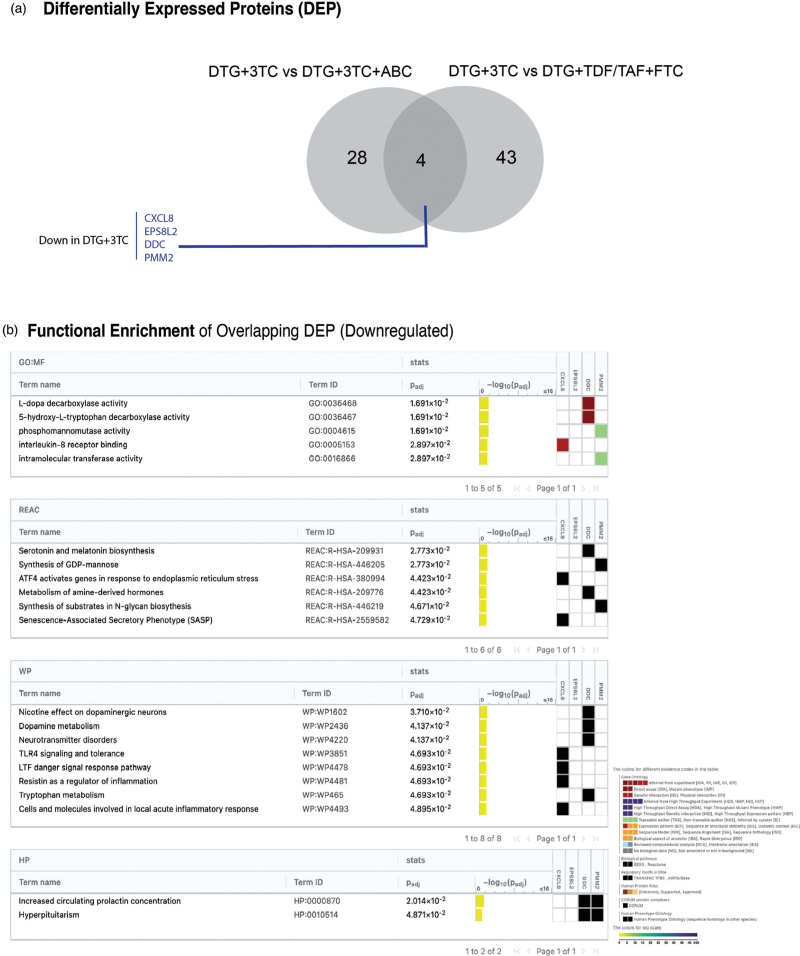
Overrepresentation of overlapping DEP between PWH using 2DR vs. 3DR.

Across all 2DR vs. 3DR comparisons, four DEPs were consistently downregulated in 2DR: CXCL8, EPS8 signaling adaptor L2 (EPS8L2), dopa decarboxylase (DDC), and phosphomannomutase 2 (PMM2) pointing to a common molecular signature of reduced NRTI exposure (Fig. 3a). Individual analysis within the TDF/TAF 3DR group revealed that TDF was associated with the upregulation of EPS8L2 (log_2_FC: −0.5), DDC (log_2_FC: −0.8), and PMM2 (log_2_FC: −0.5) in 3DR, while TAF primarily contributed to the upregulation of CXCL8 (log_2_FC: −0.6). Overrepresentation analysis of the overlapping downregulated DEPs identified significant enrichment (FDR < 0.05) (Fig. 3b). Notably, enriched pathways (downregulated in 2DR) involved neurotransmitter biosynthesis (e.g., dopamine, L-dopa decarboxylase activity, and neurotransmitter disorders) and phosphomannomutase activity (e.g. synthesis of GDP-mannose), with DDC and PMM2 identified as leading DEPs. Immune-related pathways, such as ATF4 activation in response to endoplasmic reticulum stress, TLR4 signaling, and the LTF danger signal response pathway were also significantly enriched, with CXCL8 as key DEP.

(a) Venn diagram of the DEP found in the 2DR (DTG + 3TC) vs. 3DR (DTG + 3TC + ABC and DTG + TDF/TAF + FTC) comparisons. (b) Overrepresentation analysis results (FDR P-value < 0.05) of the four overlapping downregulated differentially expressed proteins (DEPs) in DTG + 3TC vs. DTG + 3TC + ABC and DTG + TDF/TAF + FTC, performed using g:Profiler g:GOSt with the total proteins used for differential expression analysis as the reference gene background set, Benjamini–Hochberg FDR correction, and significance threshold of 0.05.

In summary, plasma proteome highlights consistent downregulation of metabolic and immune-related pathways in the 2DR compared to both 3DR. Moreover, four proteins (CXCL8, EPS8L2, DDC, and PMM2) were identified as consistently downregulated in 2DR across the different comparisons, suggesting a common molecular signature linked to the reduction of NRTIs between 3DR and 2DR DTG-based cART regimens.

### Transcriptomics reveal downregulation of stress response and apoptosis in participants using treatments with one NRTI compared to those using two NRTIs

**Fig. 4:**
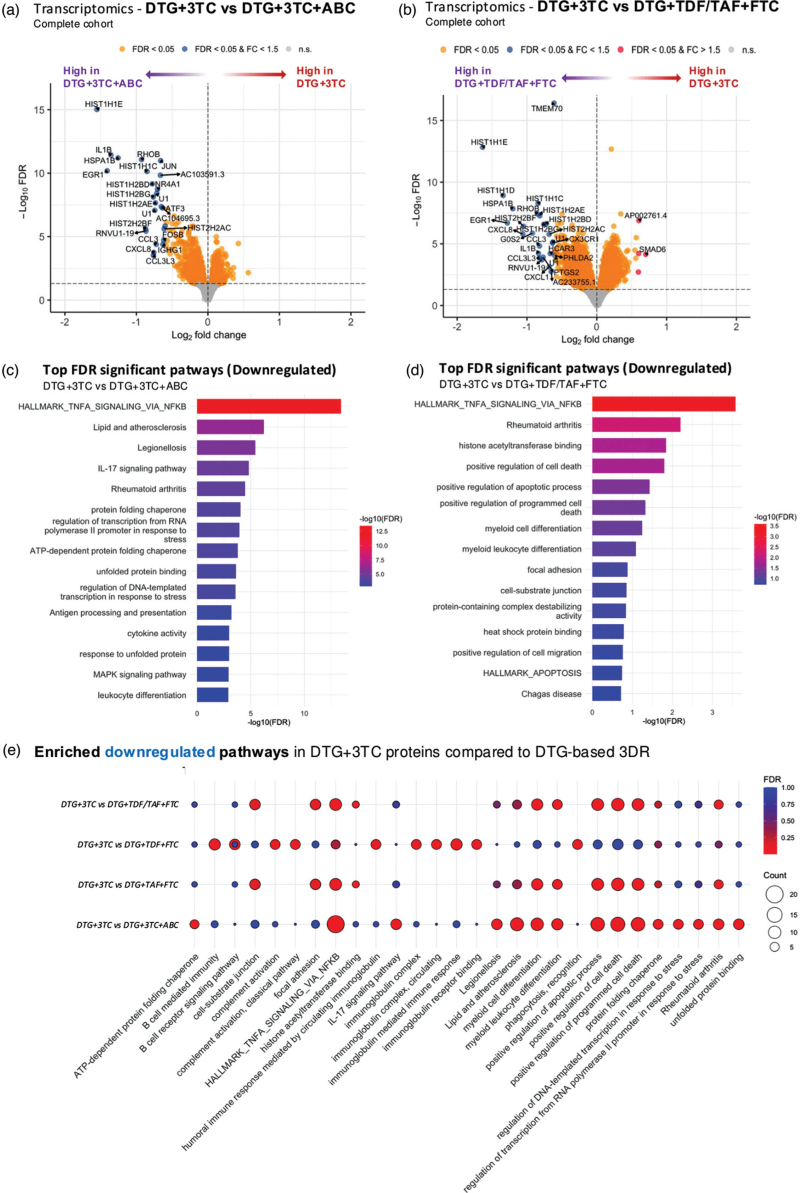
Differential gene expression and pathway enrichment in PWH using 2DR and 3DR.

First, we compared gene expression between 2DR: DTG + 3TC to 3DR: DTG + 3TC + ABC and found 25 downregulated (log_2_FC: −1.56 to −0.60, FDR < 0.05) DEGs in 2DR (Fig. 4a). Pathway analysis revealed significant enrichment in several pathways downregulated in 2DR, including TNFα signaling via NF-κB (Hallmark), lipid metabolism and atherosclerosis (hsa05417), IL-17 signaling pathway (hsa04657), and stress-related transcriptional regulation pathways such as regulation of transcription from RNA polymerase II promoter in response to stress (GO:0043618), ATP-dependent protein folding chaperone (GO:0140662), and regulation of DNA-templated transcription in response to stress (GO:0043620), hypoxia, and apoptosis (FDR < 0.05) as shown in Tables S6 and S12, Supplemental Digital Content 1.

(a, b) Volcano plots of the differentially expressed genes between PWH under DTG + 3TC vs. DTG + 3TC + ABC (a) and DTG + TDF/TAF + FTC (b). X-axis depicts Log_2_ fold change of differentially expressed genes and Y-axis depicts the −Log_10_(FDR *P* value). Differential expression analysis was performed using age, sex, plate, and center (RUMC) as covariates. (c, d) Barplot with the top 15 most FDR *P*-value significant enriched pathways by the downregulated genes in the comparison between DTG + 3TC vs. DTG + 3TC + ABC (a) and DTG + TDF/TAF + FTC (b). X-axis depicts the −log_10_(FDR *P*-value) significance of the pathway and the Y-axis the pathway terms. (e) Balloon plot of the top 10 most significant (FDR *P*-value) downregulated pathways per 2DR vs. 3DR comparison. Color indicates FDR *P*-value significance and the size the count of the genes contributing to the pathway enrichment.

Next, comparing 2DR: DTG + 3TC with 3DR: DTG + TDF/TAF + FTC revealed 27 downregulated (log_2_FC: −1.64 to −0.59) and 4 upregulated DEGs (log_2_FC: 0.60–0.70) in 2DR. Upregulated genes included SMAD family member 6 (*SMAD6*), Synemin (*SYNM*), prostaglandin E synthase (*PTGES*), and *AP002761.4* (Fig. 4b). Pathway enrichment analysis of the most significant downregulated genes highlighted several pathways related to apoptosis and inflammation such as TNFα signaling via NF-κB (Hallmark), leukocyte activation involved in inflammatory response (GO:0002269), and positive regulation of cell death (GO:0010942) with FDR <0.05 (Tables S7 and S13, Supplemental Digital Content 1).

To further explore the individual effects of TDF and TAF as NRTIs, we compared 2DR with 3DR regimens using either TDF or TAF. In the comparison between 2DR: DTG + 3TC and 3DR: DTG + TDF + FTC, 23 downregulated DEGs (log_2_FC: −1.71 to −0.60) were identified in 2DR, and significant enrichment was observed in pathways (downregulated in 2DR) related to complement activation via the classical pathway (GO:0006958), B cell receptor signaling (GO:0050853), and humoral immune response mediated by circulating immunoglobulin (GO:0002455). In contrast, 2DR: DTG + 3TC compared with 3DR: DTG + TAF + FTC revealed 62 downregulated (log_2_FC: −1.96 to −0.60) and 15 upregulated (log_2_FC: 0.6–1.12) DEGs in 2DR, with similar pathway enrichment of apoptotic and cell death processes lower in 2DR as observed in 2DR: DTG + 3TC vs. 3DR: DTG + TDF/TAF + FTC (Fig. S4 and Tables S8-9 and S14–S15, Supplemental Digital Content 1).

**Fig. 5:**
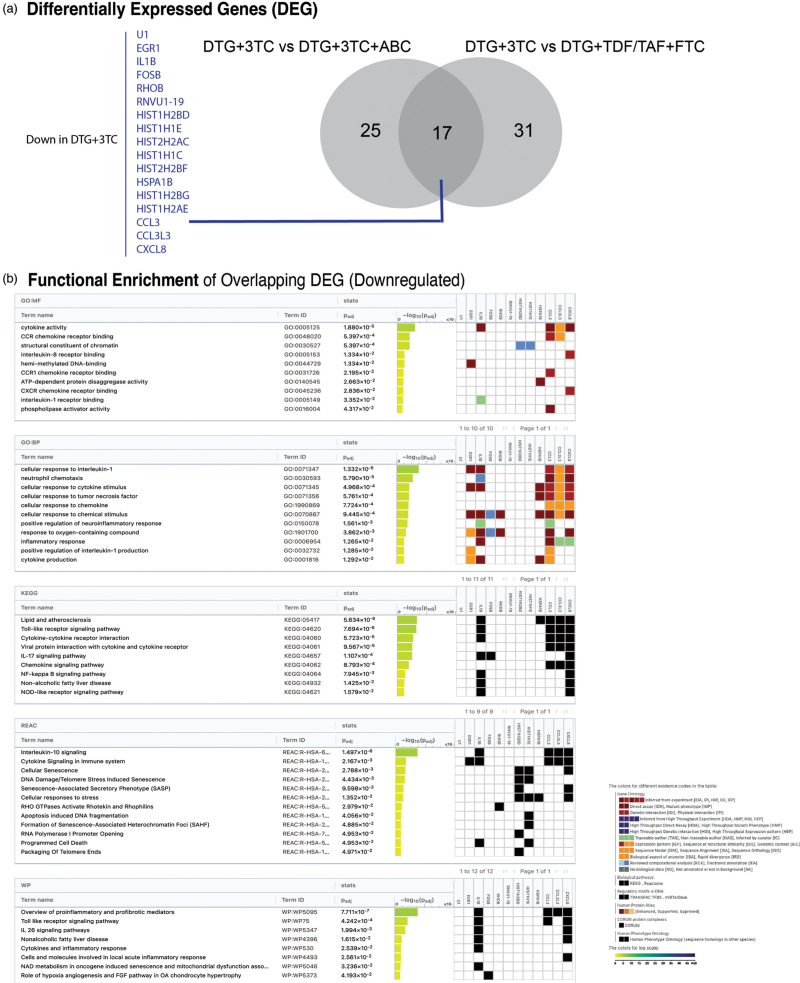
Overrepresentation of overlapping DEG between PWH using 2DR vs. 3DR.

Overall, we identified 17 DEGs consistently downregulated in 2DR compared to 3DR, consistent before and after FDR adjustment, detailed in Fig. 5a. CXCL8 was a common downregulated marker in proteome and transcriptome layers (Fig. S5, Supplemental Digital Content 1). Overrepresentation analysis revealed significant enrichment (FDR<0.05) in pathways related to chromatin structure, nucleosome assembly, cellular senescence, stress response, and DNA damage/telomere stress-induced senescence, with *HIST1H2BD*, *HIST1H1E*, and *CXCL8* as key genes (Fig. 5b). Additional enriched pathways included hemi-methylated DNA binding and lipid biosynthesis regulation, with *EGR1* as a key DEG. Pathways related to cytokine activity, cellular response to IL-1, and signaling pathways (IL-17, Toll-like receptor, NF-kappa B, NOD-like receptor, and cytokine-cytokine receptor interaction) and senescence such as NAD metabolism in oncogene induced senescence and mitochondrial dysfunction associated senescence were also enriched, with *IL1B* and *CXCL8* identified as leading DEGs.

(a) Venn diagram of the DEG found in the 2DR (DTG + 3TC) vs. 3DR (DTG + 3TC + ABC and DTG + TDF/TAF + FTC) comparisons. (b) Overrepresentation analysis results (FDR *P*-value < 0.05) of the 17 overlapping downregulated differentially expressed genes (DEGs) in DTG + 3TC vs. DTG + 3TC + ABC and DTG + TDF/TAF + FTC, performed using g:Profiler g:GOSt with the total genes used for differential expression analysis as the reference gene background set, Benjamini–Hochberg FDR correction, and significance threshold of 0.05.

### Ex-vivo cytokine analysis reveals no major differences across treatment groups

Ex-vivo cytokine production capacity of PBMCs was assessed to determine if observed downregulation of immune-related pathways observed in the proteomics and transcriptomics translated into altered immune cell function. No statistically significant differences (FDR < 0.05) were found between treatment groups for the 24-h stimulation. During the 7-day stimulation, a statistically significant downregulation of IL-17 was observed with *C. albicans hyphae* and *S. aureus* in the 2DR: DTG + 3TC group compared to 3DR: DTG + TDF + FTC. Other comparisons also indicated a trend towards downregulation of IL-17, although these results did not achieve FDR significance (Figs. S2 and S6, Supplemental Digital Content 1).

Results across all layers remained consistent after sensitivity analyses; see Figs. S7–S10, Supplemental Digital Content 1.

## Discussion

Our study shows that reducing NRTIs in cART regimens is associated with decreased activation of stress response and immune-related pathways, without compromising the functional capacity of circulating immune cells, suggesting that DTG-based 2DR regimens may be associated with a distinct immunological profile compared to 3DR in PWH.

Plasma proteomics, giving insight into systemic biological processes, revealed significant downregulation of pathways involved in metabolism, stress response, and immune signaling in 2DR users compared to 3DR. Notably, only four proteins – CXCL8, DDC, PMM2, and EPS8L2 – were consistently downregulated across comparisons. Elevated CXCL8 (IL-8) is a hallmark of chronic inflammation in PWH on ART, linked to morbidity and mortality [30] and its blockade has been suggested as possible therapeutic option in HIV-related neuroinflammatory processes [31]. Its downregulation in 2DR suggests reduced systemic inflammation. DDC has been implicated in several neurotransmitter disorders, including Parkinson's disease and hypertension [32]. PMM2, a glycosylation enzyme, could influence immune cell function [33], while EPS8L2 has been linked to neurotoxicity in cancer therapeutics [34]. These changes suggest differences in immune modulation between regimens.

Comparing the effect of TAF and TDF in 3DR vs. 2DR, TAF use was associated to CXCL8 upregulation, while TDF was linked to more DEPs, highlighting potential differences in immune activation and stress modulation between regimens.

Different studies show 2DR and 3DR are equally effective and safe [7,35,36], but their influence on systemic inflammation varies, with some reporting no impact on biomarkers [37], while others suggest anti-inflammatory benefits [38].

Gene expression analysis of PBMCs, providing insights into transcriptional changes in circulating immune cells, corroborated these findings, showing predominantly downregulated genes in 2DR compared to 3DR, which may differ between the NRTIs that used in the 3DR. However, there are 17 overlapping downregulated genes across all 3DR vs. 2DR comparisons and the related downregulated pathways show an association with chromatin structure, cellular senescence, stress response, and cytokine activity pathways. Remarkably, gene expression profiles showed more cell death and senescence in TDF/TAF users compared to ABC, with TAF driving greater gene upregulation than TDF, despite its milder effect on protein expression. These findings, along with reduced activation of stress and immune pathways in 2DR compared to 3DR, highlight the complex biological responses to different NRTIs across proteomic and transcriptomic levels.

Despite these molecular changes, the functional capacity of immune cells, evidenced by cytokine production, remained largely unchanged between 2DR and 3DR. This aligns with previous studies showing no significant differences in immune activation markers between regimens [39]. Our ex vivo stimulation assays confirmed similar monocyte and lymphocyte functionality [40,41], except for an enhanced IL-17 production capacity associated with TDF use in 3DR. IL-17's role in stimulating IL-8 production highlights a potential interaction that may contribute to inflammatory processes in PWH [42]. Moreover, we previously showed that IL-17 production capacity of PBMC of PWH using cART or healthy controls was not different [43].

Overall, our findings indicate NRTI reduction in 2DR preserves immune function while reducing cellular stress, potentially optimizing immune balance in PWH. However, the clinical significance of these immunologic differences remains uncertain and requires further investigation. Bailón *et al.*'s findings on comparable viral reservoir reductions support 2DR and 3DR equivalence in controlling immune activation and viral reservoirs [39].

Our study has limitations, including its cross-sectional design, which prevents causal conclusions, and we did not assess tissue-specific effects beyond plasma and PBMCs. The inclusion of 3TC in the 2DR and FTC in the 3DR may have influenced results, though the data did not allow for this analysis. Additionally, data on participants’ cART history and the rationale behind treatment prescriptions were unavailable. Nevertheless, the strengths of our study are substantial. It represents one of the most comprehensive proteomics and transcriptomics analyses comparing DTG-based 2DR and 3DR, with 494 participants, over 50.000 gene transcripts, and 2.367 proteins, confirming results across various omics layers.

Our findings expand upon previous research highlighting the lower toxicity of DTG-based 2DR regimens. Li *et al.* reported higher mitochondrial DNA mutations in NRTI-based cART, while DTG+3TC regimens have shown reduced mitochondrial dysfunction [44–46]. This aligns with our findings showing reduced activation of stress and immune pathways in 2DR, which may reflect diminished mitochondrial stress, contributing to the long-term safety of 2DR regimens.

ABC use in cART has been linked to increased CVD risk, though the mechanisms are unclear [47]. Our study reveals downregulation of lipid metabolism, atherosclerosis, and metabolic pathways in 2DR compared to 3DR containing ABC. This suggests a potential mechanism for ABC's association with CVD risk, as metabolic dysregulation has been linked to atherosclerosis, the main underlying cause of CVD [48,49]. The reduced activation of these pathways in 2DR indicates it may lower CVD risk by reducing lipid dysregulation, emphasizing the benefits of decreased NRTI exposure.

The impact of cART on immune activation and senescence is crucial for understanding long-term health in PWH. Prolonged exposure to cART, particularly regimens with multiple NRTIs, can increase inflammation, immune dysregulation, and T-cell senescence [50–53]. Additionally, the interplay between oxidative stress and telomere dynamics is noteworthy. Oxidative stress accelerates telomere shortening, contributing to cellular aging and senescence [54]. Our observation of reduced immune and oxidative stress-related pathways in 2DR may support telomere integrity, potentially improving cellular longevity in PWH. This aligns with studies showing improved telomere maintenance in individuals switching from 3DR to 2DR [45,55].

In conclusion, our findings reveal that DTG-based 2DR regimens may be associated with a more favorable immunological profile than 3DR regimens by potentially minimizing long-term adverse effects of NRTI-related chronic immune activation, cellular senescence, stress responses, and mitochondrial toxicity. However, these findings should be interpreted with caution, as their clinical significance remains unclear. Further longitudinal and functional studies are needed to translate these findings into meaningful clinical outcomes. Importantly, we did not observe increased inflammatory markers in the 2DR group. These results support the use of DTG-based 2DR as an optimized treatment strategy for PWH without prior treatment failure or HBV co-infection, though further research is needed to confirm its clinical relevance.

## Acknowledgements

Our extensive gratitude goes to all the volunteers who participated in this study. In addition, we would like to thank all those who helped recruiting participants and contributed in the laboratory. Also, we would like to show our appreciation to all physicians and nurses who helped in informing potential participants in OLVG, ErasmusMC, ETZ and Radboudumc. In addition, authors acknowledge the effort and contribution of the clinical and laboratory staff who helped with participants recruiting and samples processing.

Authors contribution: V.R. performed the statistical analyses and wrote the manuscript. W.V., A.V., and J.L. wrote sections of the manuscript and gave critical revision. W.V., A.G., M.B., and L.E. collected data at baseline. L.J., M.N., and A.V. designed the study and oversaw the project. All authors contributed to manuscript revision, read, and approved the submitted version.

### Conflicts of interest

All authors are part of the 2000HIV collaboration, supported by ViiV Healthcare. This research was funded by ViiV Healthcare. However, ViiV Healthcare had no involvement in data quality control, statistical analyses, or the final interpretation of the results.

## Supplementary Material
