# Supplementary material for: Dolutegravir plus lamivudine downregulates cellular stress responses vs. three-drug HIV regimens
Source: AIDS. 2025 Apr 1;39(9):1106–19. doi: 10.1097/QAD.0000000000004198 (PMC12237123; doi:10.1097/QAD.0000000000004198)
Supplement: Supplementary file 1 [file aids-39-1106-s001.docx]

**Supplementary Material**

**Table S1.** Baseline Clinical Characteristics of PLHIV under DTG-based 2DR and 3DR in the 2000HIV study

|  | **DTG + 3TC** | **DTG + 3TC + ABC** | **DTG + TDF/TAF + FTC** | **p.overall** | **N** |
| --- | --- | --- | --- | --- | --- |
|  | ***N=191*** | ***N=188*** | ***N=115*** |  |  |
| **Demographics** |  |  |  |  |  |
| Age | 53.0 [44.0;60.0] | 50.0 [41.0;59.2] | 49.0 [39.5;55.5] | 0.010 | 494 |
| Sex at birth (Male) | 164 (85.9%) | 157 (83.5%) | 102 (88.7%) | 0.455 | 494 |
| Ethnicity: |  |  |  | 0.786 | 494 |
| Asian | 7 (3.66%) | 6 (3.19%) | 8 (6.96%) |  |  |
| Black | 17 (8.90%) | 19 (10.1%) | 12 (10.4%) |  |  |
| Hispanic | 4 (2.09%) | 6 (3.19%) | 5 (4.35%) |  |  |
| Mixed | 12 (6.28%) | 8 (4.26%) | 6 (5.22%) |  |  |
| Native American | 0 (0.00%) | 1 (0.53%) | 0 (0.00%) |  |  |
| White | 151 (79.1%) | 147 (78.2%) | 84 (73.0%) |  |  |
| Smoking (yes) | 57 (29.8%) | 42 (22.3%) | 41 (35.7%) | 0.141 | 494 |
| Statins use in the last two weeks (yes) | 33 (17.3%) | 36 (19.1%) | 24 (20.9%) | 0.731 | 494 |
| Intravenous drugs use (yes) | 1 (0.52%) | 2 (1.06%) | 1 (0.87%) | 0.840 | 494 |
| **HIV** |  |  |  |  |  |
| HIV duration (years) | 10.5 [5.51;15.8] | 9.20 [5.29;14.9] | 7.01 [2.52;14.7] | 0.018 | 494 |
| HIV stage (CDC): |  |  |  | . | 494 |
| 0 | 35 (18.3%) | 34 (18.1%) | 18 (15.7%) |  |  |
| 1 | 95 (49.7%) | 102 (54.3%) | 47 (40.9%) |  |  |
| 2 | 60 (31.4%) | 47 (25.0%) | 49 (42.6%) |  |  |
| 3 | 1 (0.52%) | 5 (2.66%) | 1 (0.87%) |  |  |
| CD4 pre-cART  (10^9 cells/L) | 0.31 [0.19;0.48] | 0.33 [0.23;0.50] | 0.30 [0.14;0.42] | 0.155 | 482 |
| CD4 nadir  (10^9 cells/L) | 0.29 [0.18;0.44] | 0.32 [0.21;0.43] | 0.27 [0.12;0.42] | 0.138 | 488 |
| CD4 latest  (10^9 cells/L) | 0.71 [0.56;0.85] | 0.76 [0.56;0.95] | 0.66 [0.47;0.86] | 0.016 | 487 |
| Viral load pre-cART (copies/mL) | 69414 [18533;179000] | 78200 [28988;200000] | 87394 [27300;200000] | 0.385 | 464 |
| Viral load zenith (copies/mL) | 88600 [25500;200000] | 100000 [39560;286500] | 100000 [42813;300000] | 0.179 | 464 |
| Quantifiable viral load (copies/mL) | 0.00 [0.00;49.8] | 63.5 [12.2;94.2] | 40.0 [0.00;40.0] | 0.180 | 29 |
| **ART** |  |  |  |  |  |
| cART duration (years) | 7.91 [4.53;12.9] | 7.31 [4.77;12.8] | 6.07 [2.29;11.5] | 0.026 | 494 |
| Early cART (Did participant initiate cART <1 month after confirmed acute HIV infection) | 12 (6.28%) | 15 (7.98%) | 12 (10.4%) | 0.001 | 494 |
| History of cART interruption (>3 Months) | 30 (15.7%) | 19 (10.1%) | 13 (11.3%) | 0.232 | 494 |
| **Co-infections** |  |  |  |  |  |
| CMV IgG serology (positive) | 175 (91.6%) | 173 (92.0%) | 104 (90.4%) | 0.896 | 494 |
| Previous HEPB infection (yes) | 69 (36.1%) | 58 (30.9%) | 29 (25.2%) | 0.117 | 494 |
| Previous HEPB vaccination (yes) | 98 (51.3%) | 98 (52.1%) | 53 (46.1%) | 0.338 | 494 |
| HEPC infection (yes) | 15 (7.85%) | 15 (7.98%) | 10 (8.70%) | 0.964 | 494 |
| Treated HEPC infection (yes) | 15 (7.85%) | 15 (7.98%) | 9 (7.83%) | 0.699 | 494 |
| COVID19 infection | 30 (15.7%) | 19 (10.1%) | 13 (11.3%) | 0.232 | 494 |
| COVID vaccinated (yes) | 59 (30.9%) | 41 (21.8%) | 20 (17.4%) | 0.002 | 494 |
| **Comorbidities** |  |  |  |  |  |
| Osteoporosis diagnosed (yes) | 7 (3.66%) | 5 (2.66%) | 1 (0.87%) | 0.370 | 494 |
| Osteoarthritis diagnosed (yes) | 4 (2.09%) | 6 (3.19%) | 5 (4.35%) | 0.504 | 494 |
| Peripheral arterial vascular disease diagnosed (yes) | 1 (0.52%) | 1 (0.53%) | 4 (3.48%) | 0.057 | 494 |
| Cardiovascular disorder diagnosed (yes) | 64 (33.5%) | 60 (31.9%) | 31 (27.0%) | 0.479 | 494 |

Baseline clinical characteristics for the 2000HIV cohort. Statistical analyses were performed using the Kruskal-Wallis test for numerical variables and the Chi-squared test for categorical variables. Categorical variables are presented as n (%), and numerical variables are expressed as Median [Q1;Q3] due to the non-normal distribution of values. Previous Hepatitis B infection was indicated by the presence of anti-HBc antibodies, whereas previous Hepatitis B vaccination was indicated by the presence of anti-HBs antibodies in the medical history. Previous Hepatitis C infection was indicated by positive HCV regardless of treatment or spontaneous clearance. Quantifiable viral load was reported for detectable cases (>40 copies/mL). ART Interruption (>3 Months) Indicates if ART was interrupted for more than three consecutive months after treatment initiation.

**Figure S1
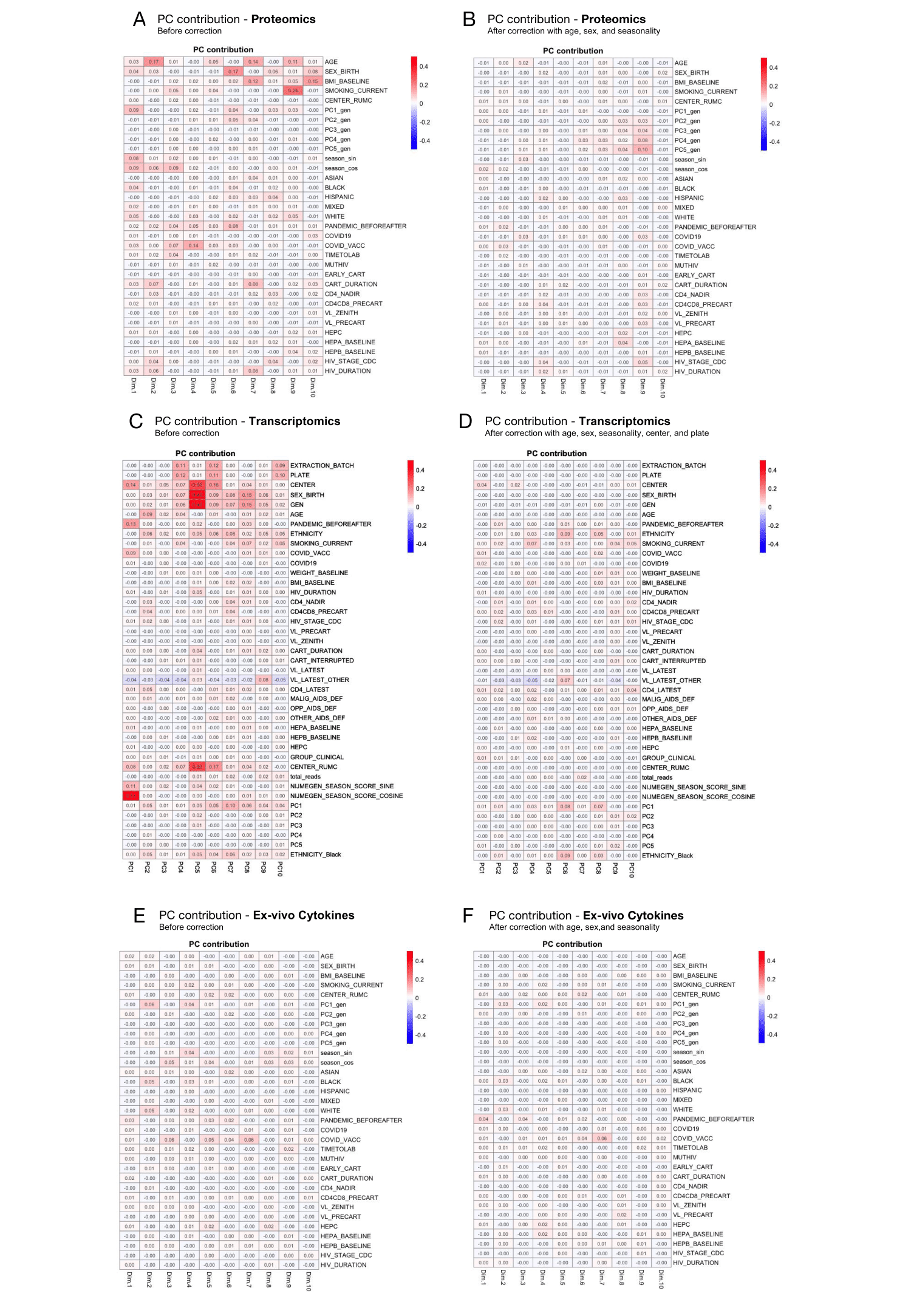
**

**Figure S1. Principal components contribution across *omics* layers**

**A-F** Heatmaps depicting the associations between first 10 principal components (PCs) of omics data and list of possible confounders. The color-coding key depicts the beta estimate calculated by a linear regression model. Potential confounding variables were defined as those that, when added to the linear model in a multi-step approach, resulted in more than a 10% increase in beta coefficients.

**A,C,E** show associations in the uncorrected data, whereas **B,D,F** associations after correcting for age, sex, center (RUMC), plate, and seasonality scores.

**Figure S2**

**
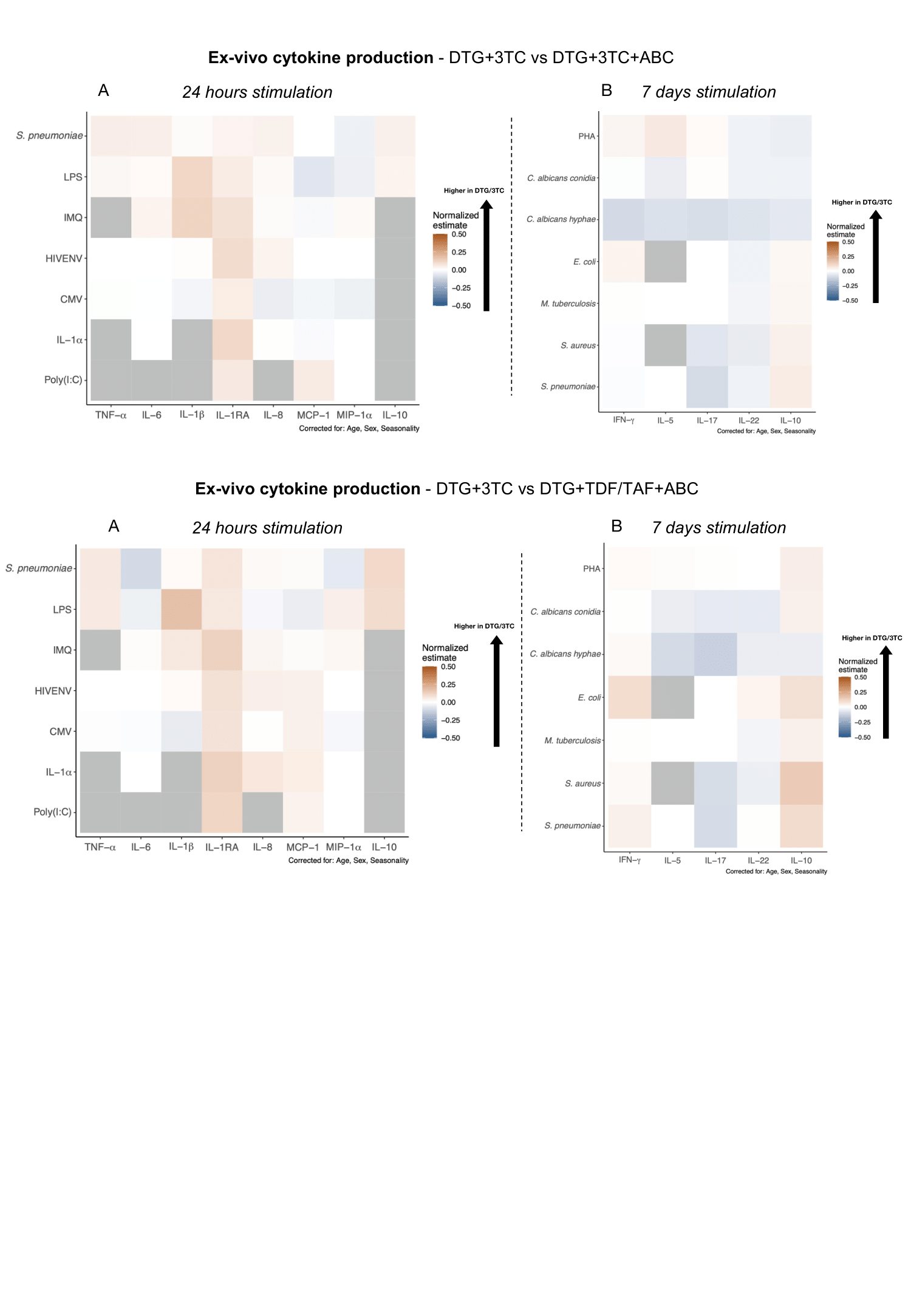
**

D

C

**Figure S2. Differential ex-vivo cytokine production capacity across 2DR vs 3DR users**

**A,C.** Ex-vivo cytokine production levels. Results for 24 hours stimulation where Pvalue <= 0.0005 is "***", Pvalue <= 0.005 is “**”, and Pvalue <= 0.05 is “*”.

**B,D.** Results for 7 days stimulation where Pvalue <= 0.0005 is "***", Pvalue <= 0.005 is “**”, and Pvalue <= 0.05 is “*”.

**Figure S3**


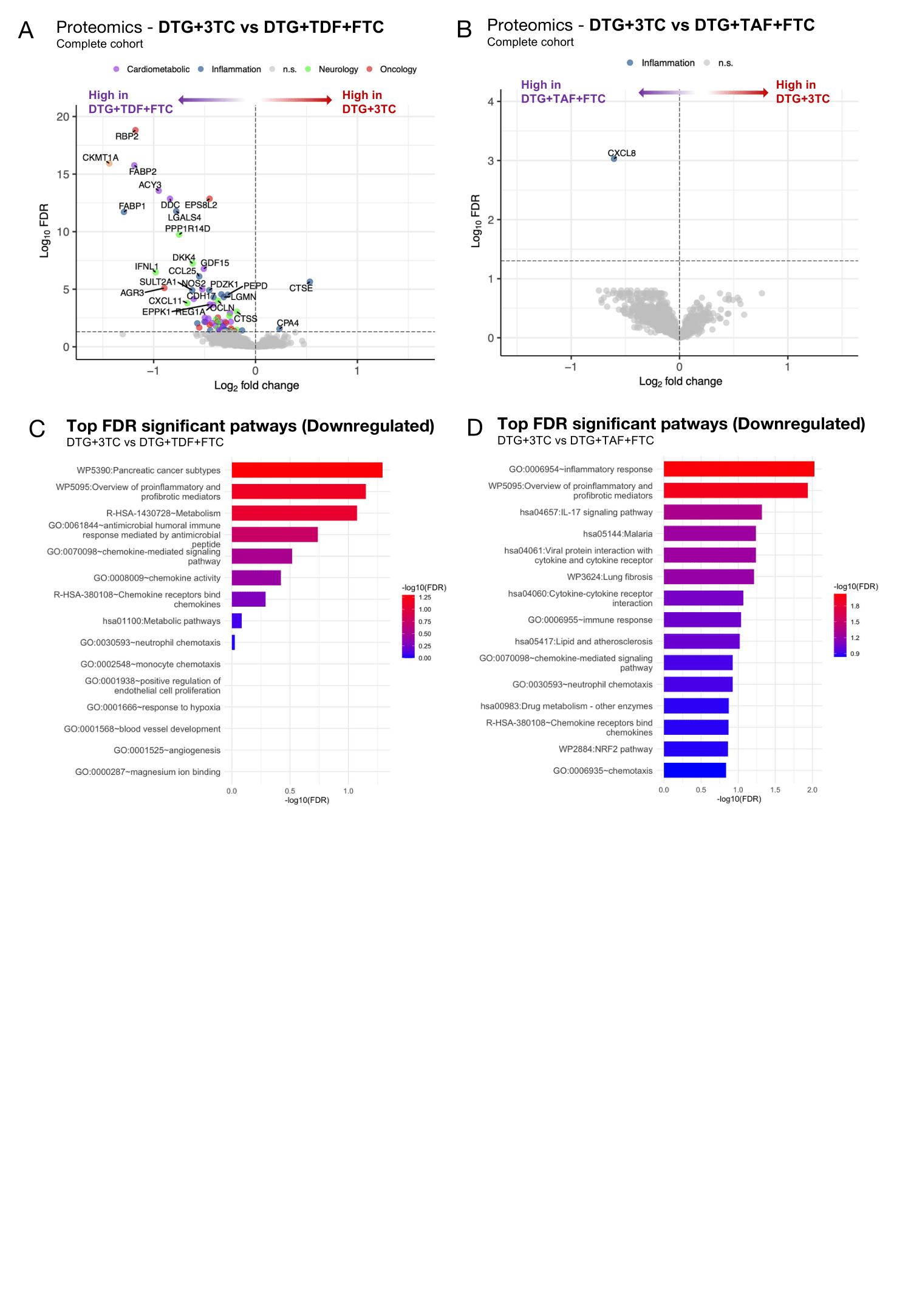


**Figure S3. Differential protein expression between 2DR vs DTG + TDF + FTC and DTG+TAF+FTC**

**A,B.** Volcano plots of the differentially expressed proteins between PLHIV under DTG + 3TC vs DTG + TDF + FTC (**A**) and DTG+TAF+FTC (**B**). X-axis depicts Log2 fold change of differentially expressed proteins and Y-axis depicts the -Log10(FDR P value). Differential expression analysis was performed using age, sex, plate and center (RUMC) as covariates.

**Figure S4**


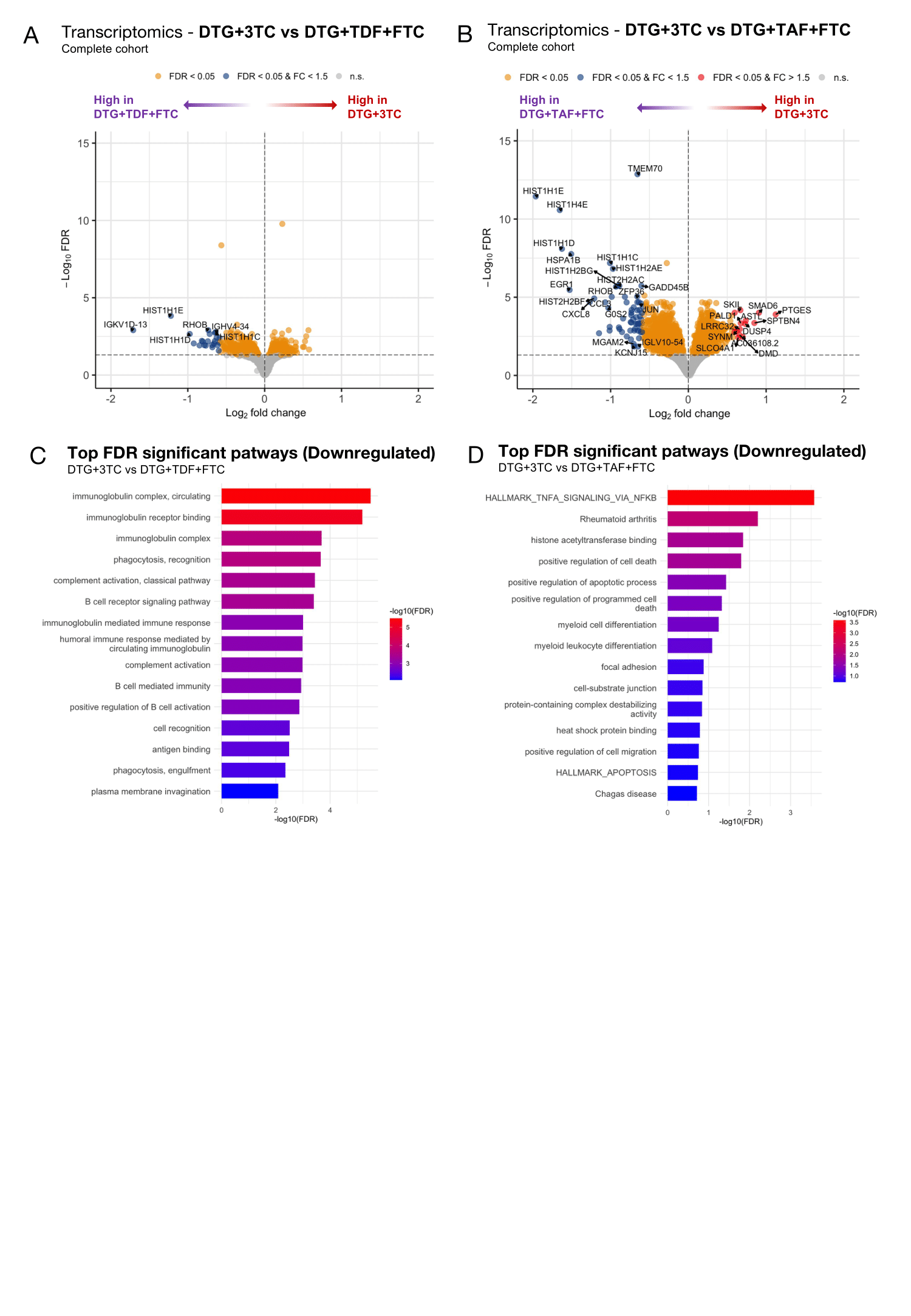


**Figure S4. Differential gene expression and pathway enrichment between 2DR vs DTG + TDF + FTC and DTG+TAF+FTC**

**A,B.** Volcano plots of the differentially expressed genes between PLHIV under DTG + 3TC vs DTG + TDF + FTC (**A**) and DTG+TAF+FTC (**B**). X-axis depicts Log2 fold change of differentially expressed genes and Y-axis depicts the -Log10(FDR P value). Differential expression analysis was performed using age, sex, plate and center (RUMC) as covariates.

**C.D.** Barplot with the top 15 most FDR P-value significant enriched pathways by the downregulated genes in the comparison between DTG + 3TC vs DTG + TDF + FTC (**A**) and DTG+TAF+FTC (**B**). X axis depicts the -log10(FDR P-value) significance of the pathway and the Y-axis the pathway terms.

**Figure S5**

**
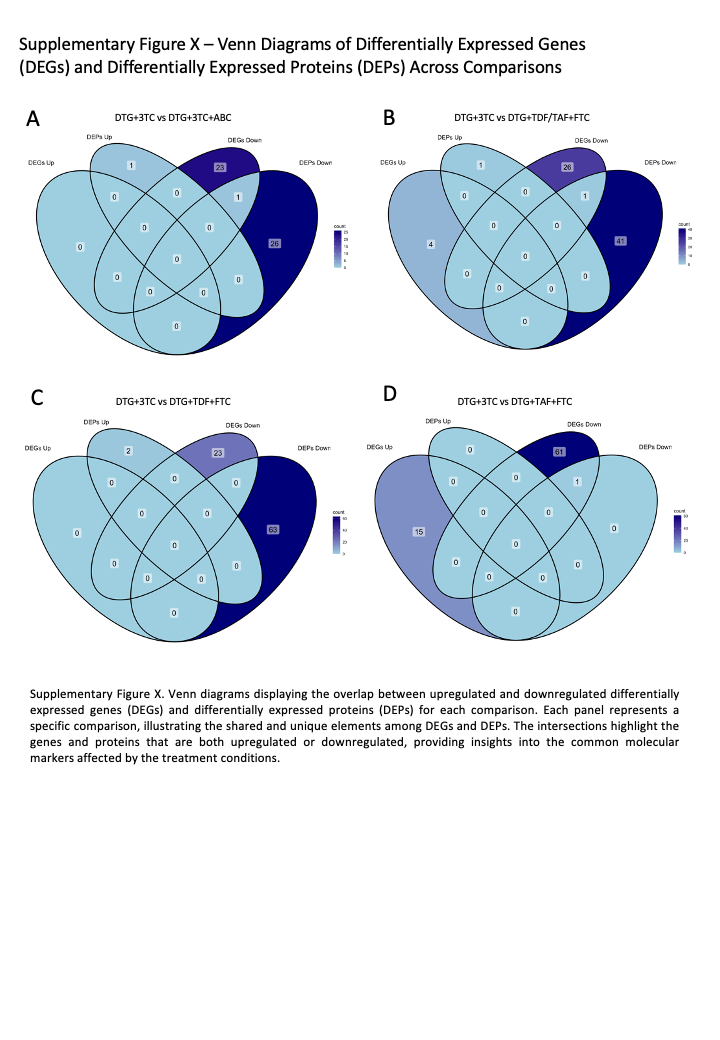
**

**Figure S5. Venn Diagrams of Differentially Expressed Genes (DEGs) and Differentially Expressed Proteins (DEPs) Across Comparisons**

Venn diagrams displaying the overlap between upregulated and downregulated differentially expressed genes (DEGs) and differentially expressed proteins (DEPs) for each comparison. Each panel represents a specific comparison, illustrating the shared and unique elements among DEGs and DEPs. The intersections highlight the genes and proteins that are both upregulated or downregulated, providing insights into the common molecular markers affected by the treatment conditions.

**Figure S6**


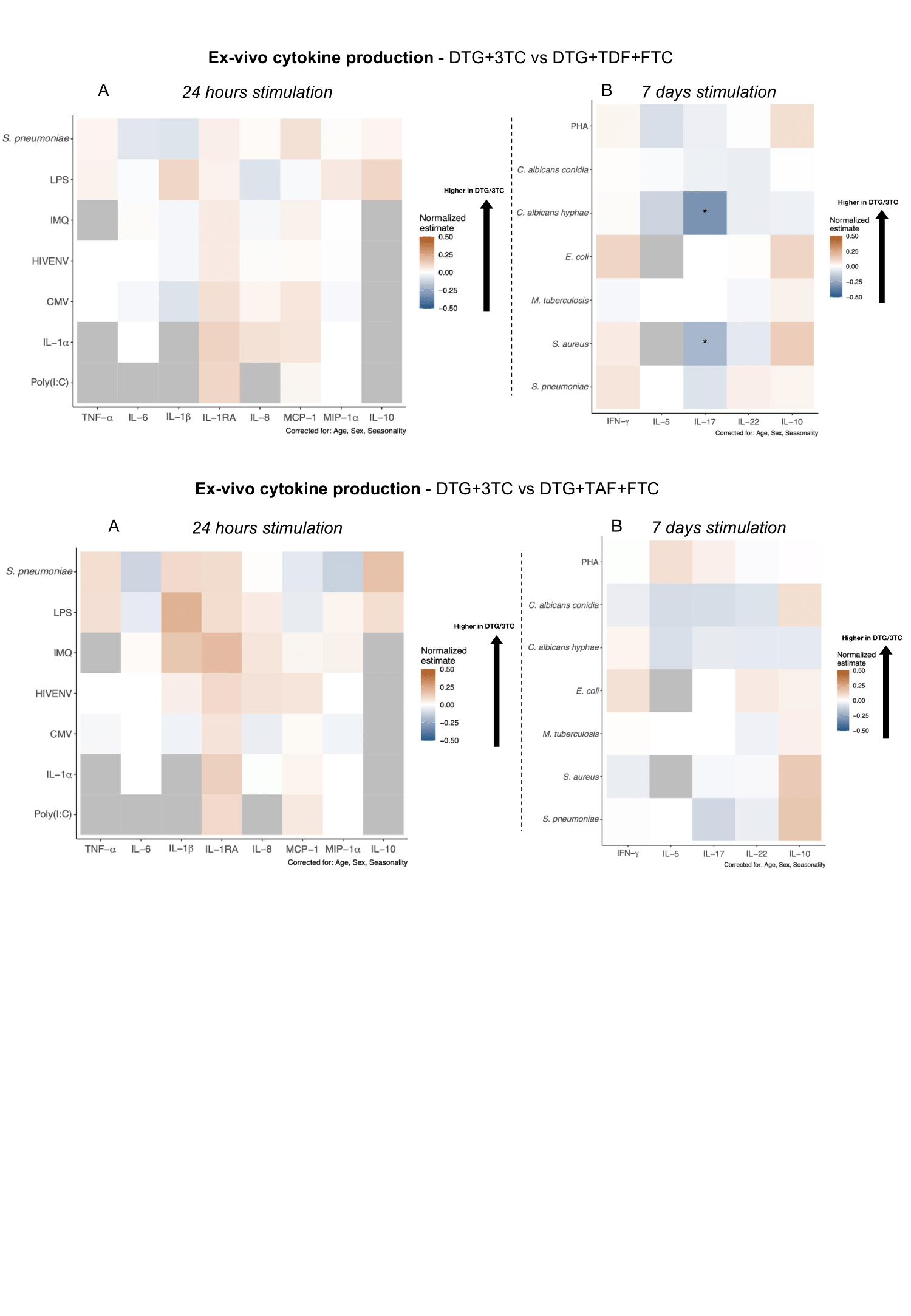


D

C

**Figure S6. Differential ex-vivo cytokine production capacity between 2DR vs DTG + TDF + FTC and DTG+TAF+FTC**

**A,C.** Ex-vivo cytokine production levels. Results for 24 hours stimulation where Pvalue <= 0.0005 is "***", Pvalue <= 0.005 is “**”, and Pvalue <= 0.05 is “*”.

**B,D.** Results for 7 days stimulation where Pvalue <= 0.0005 is "***", Pvalue <= 0.005 is “**”, and Pvalue <= 0.05 is “*”.

**Figure S7**

**
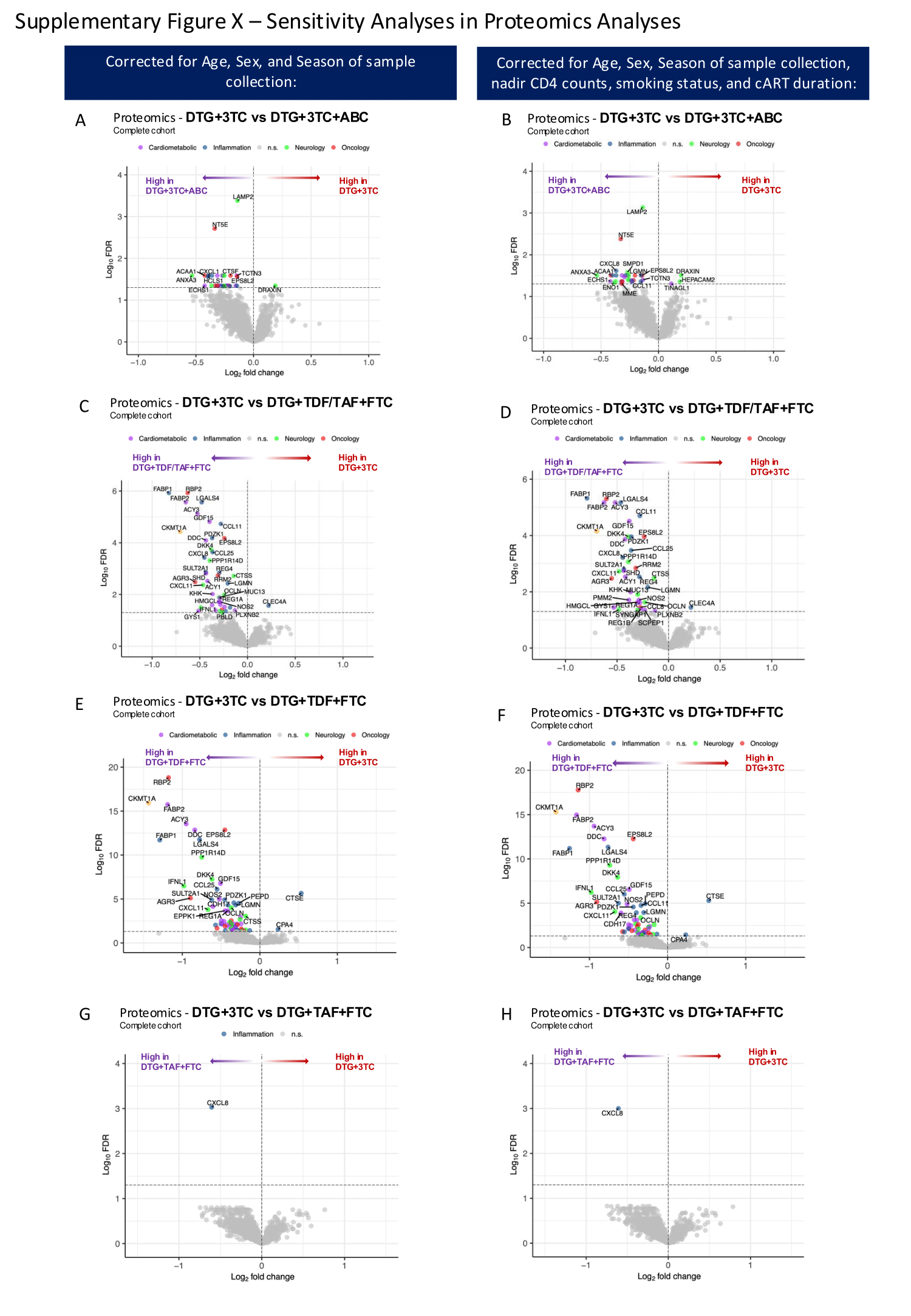
**

**Figure S7. Sensitivity analyses on proteome (Olink Explore) data**

**A-H.** Volcano plots showing differentially expressed proteins between PLHIV on 2DR vs. 3DR regimens. The x-axis represents the Log₂ fold change, and the y-axis represents the -Log₁₀(FDR P-value). Colors indicate functional categories of proteins based on the Olink Explore panels.

**A, C, E, G.** Differential expression analysis adjusted for age, sex, season of sample collection.

**B, D, F, H.** Sensitivity analyses with additional adjustments for nadir CD4 counts, smoking status, and cART duration.

**Figure S8**

**
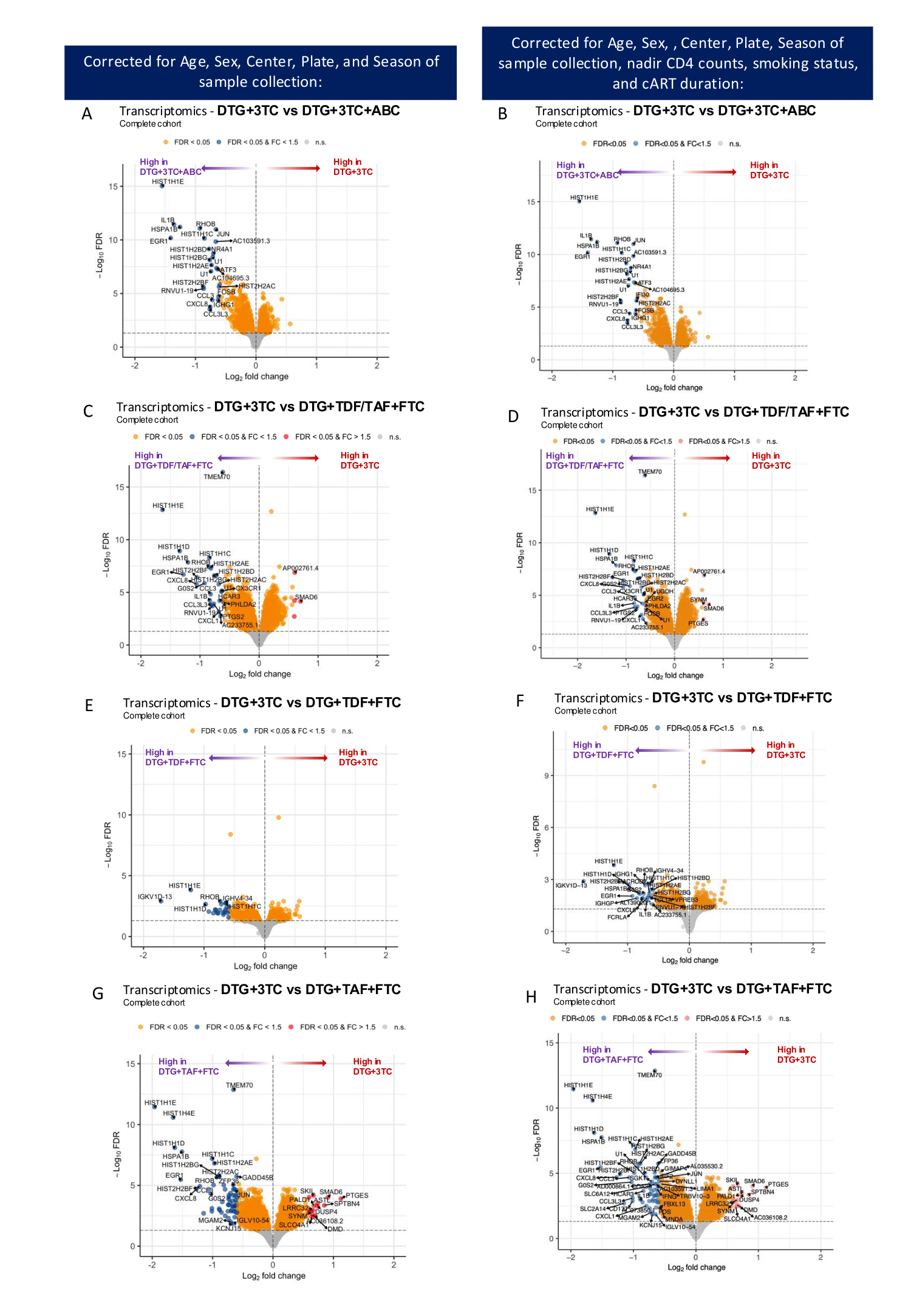
**

**Figure S8. Sensitivity analyses on transcriptome (bulk RNA-seq) data**

**A-H.** Volcano plots showing differentially expressed proteins between PLHIV on 2DR vs. 3DR regimens. The x-axis represents the Log₂ fold change, and the y-axis represents the -Log₁₀(FDR P-value). Colors indicate transcript significance and effect sizes categories.

**A, C, E, G.** Differential expression analysis adjusted for age, sex, plate, season of sample collection, and center (RUMC).

**B, D, F, H.** Sensitivity analyses with additional adjustments for nadir CD4 counts, smoking status, and cART duration.

**Figure S9**

**
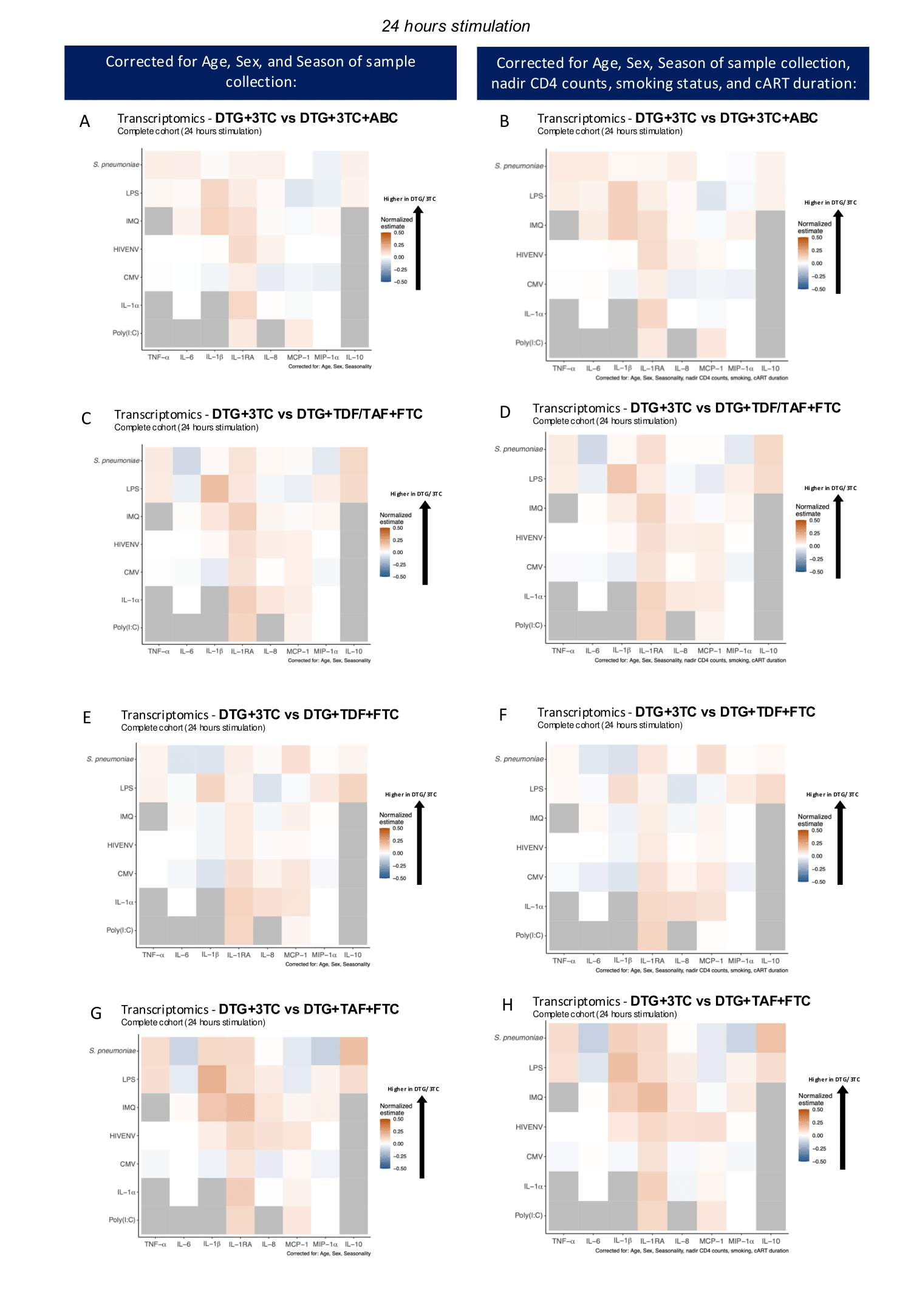
**

**Figure S9. Sensitivity Analysis of Ex-Vivo Cytokine Production Capacity Between 2DR vs 3DR After 24 Hours of Stimulation**

**A, C, E, G.** Ex-vivo cytokine production levels comparing DTG+3TC vs. different 3DR regimens, adjusted for age, sex, and season of sample collection.

**B, D, F, H.** Sensitivity analysis with additional adjustments for nadir CD4 counts, smoking status, and cART duration.

Statistical significance is indicated as follows: *** P ≤ 0.0005, ** P ≤ 0.005, * P ≤ 0.05.

**Figure S10**

**
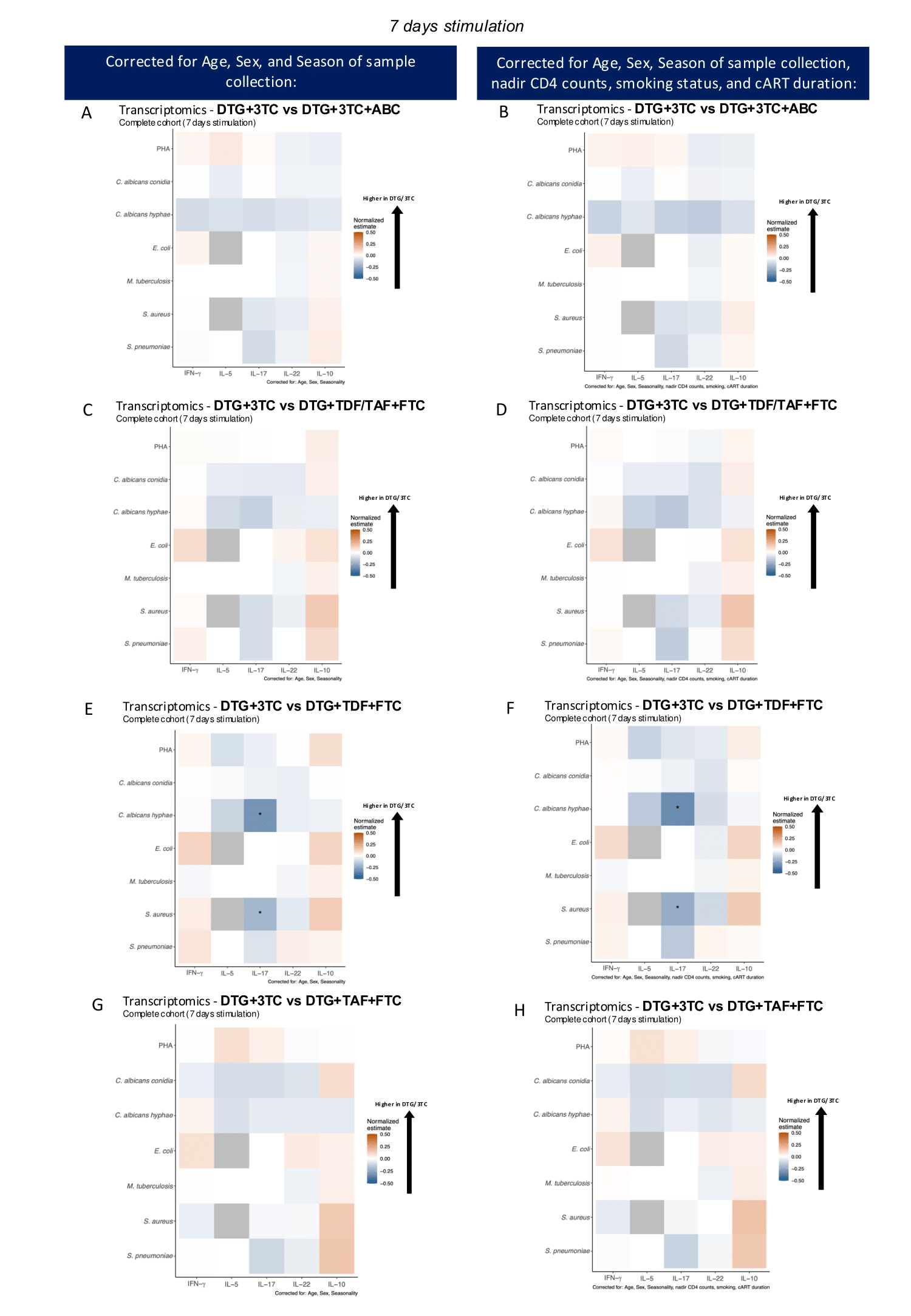
**

**Figure S10. Sensitivity Analysis of Ex-Vivo Cytokine Production Capacity Between 2DR vs 3DR After 7 Days of Stimulation**

**A, C, E, G.** Ex-vivo cytokine production levels comparing DTG+3TC vs. different 3DR regimens, adjusted for age, sex, and season of sample collection.

**B, D, F, H.** Sensitivity analysis with additional adjustments for nadir CD4 counts, smoking status, and cART duration.

Statistical significance is indicated as follows: *** P ≤ 0.0005, ** P ≤ 0.005, * P ≤ 0.05.

**Supplementary Tables**

1. **Differentially Expressed Proteins**

The differentially expressed proteins (DEP) were defined as the proteins with an FDR corrected significance (<0.05) in the complete cohort.

**Table S2.** DTG + 3TC protein levels compared to DTG + 3TC + ABC

DTG+3TC compared to 3DR: DTG+3TC+ABC identified 466 nominally significant proteins, with 27 downregulated (log2FC: -0.53 to -0.14) and one upregulated (DRAXIN, log2FC: 0.19) DEPs after FDR adjustment (FDR<0.05) in 2DR

| **Protein**  **(and Olink® Panel)** | **log FC** | **Average Expression** | **t-value** | **P-value** | **FDR** | **B-value** | **Regulation** |
| --- | --- | --- | --- | --- | --- | --- | --- |
| LAMP2_Neurology | -0.1398 | 0.3491 | -5.3420 | 1.60E-07 | 3.79E-04 | 6.8345 | down FDR |
| NT5E_Oncology | -0.3425 | -0.2571 | -4.9261 | 1.26E-06 | 1.49E-03 | 4.9924 | down FDR |
| TCTN3_Cardiometabolic_II | -0.1453 | -0.0812 | -3.8817 | 1.23E-04 | 2.45E-02 | 0.9508 | down FDR |
| EPS8L2_Oncology | -0.1469 | 0.4728 | -3.9066 | 1.11E-04 | 2.45E-02 | 1.0368 | down FDR |
| CTSF_Oncology | -0.1999 | 0.3181 | -3.9089 | 1.10E-04 | 2.45E-02 | 1.0451 | down FDR |
| SMPD1_Neurology | -0.2534 | 0.8346 | -3.8657 | 1.31E-04 | 2.45E-02 | 0.8954 | down FDR |
| GGT1_Neurology | -0.2652 | 0.3968 | -3.8575 | 1.35E-04 | 2.45E-02 | 0.8675 | down FDR |
| VAMP5_Cardiometabolic | -0.2695 | 0.7011 | -3.9438 | 9.58E-05 | 2.45E-02 | 1.1666 | down FDR |
| GRHPR_Cardiometabolic_II | -0.3173 | -0.4265 | -3.8964 | 1.16E-04 | 2.45E-02 | 1.0017 | down FDR |
| CXCL8_Inflammation | -0.3531 | 0.2556 | -4.0608 | 5.96E-05 | 2.45E-02 | 1.5824 | down FDR |
| CXCL1_Inflammation | -0.3845 | 1.8482 | -3.9639 | 8.83E-05 | 2.45E-02 | 1.2373 | down FDR |
| ACAA1_Oncology | -0.4199 | 1.2445 | -3.9584 | 9.03E-05 | 2.45E-02 | 1.2181 | down FDR |
| ANXA3_Neurology | -0.5297 | 3.2499 | -3.8829 | 1.22E-04 | 2.45E-02 | 0.9547 | down FDR |
| HCLS1_Inflammation | -0.3954 | 3.6928 | -3.7923 | 1.74E-04 | 2.94E-02 | 0.6451 | down FDR |
| DDC_Cardiometabolic | -0.2399 | 0.7014 | -3.7027 | 2.45E-04 | 3.87E-02 | 0.3457 | down FDR |
| DRAXIN_Neurology | 0.1863 | 0.8548 | 3.5245 | 4.77E-04 | 4.34E-02 | -0.2305 | up FDR |
| DCTPP1_Cardiometabolic | -0.1556 | 0.6061 | -3.5999 | 3.61E-04 | 4.34E-02 | 0.0100 | down FDR |
| IDUA_Oncology | -0.2134 | 0.7476 | -3.5487 | 4.36E-04 | 4.34E-02 | -0.1539 | down FDR |
| FOLH1_Inflammation_II | -0.2350 | 0.1603 | -3.5925 | 3.71E-04 | 4.34E-02 | -0.0139 | down FDR |
| BST2_Neurology | -0.2553 | 0.8120 | -3.5705 | 4.03E-04 | 4.34E-02 | -0.0844 | down FDR |
| NT5C_Cardiometabolic_II | -0.2894 | -0.2090 | -3.6327 | 3.20E-04 | 4.34E-02 | 0.1161 | down FDR |
| PMM2_Cardiometabolic_II | -0.3143 | -0.5277 | -3.5271 | 4.72E-04 | 4.34E-02 | -0.2226 | down FDR |
| PRDX5_Inflammation | -0.3217 | 3.7332 | -3.5359 | 4.57E-04 | 4.34E-02 | -0.1947 | down FDR |
| MME_Oncology | -0.3365 | 0.1885 | -3.6074 | 3.51E-04 | 4.34E-02 | 0.0342 | down FDR |
| CHMP1A_Neurology | -0.3694 | 2.9179 | -3.5514 | 4.32E-04 | 4.34E-02 | -0.1454 | down FDR |
| ECHS1_Cardiometabolic_II | -0.4218 | -0.1453 | -3.5421 | 4.47E-04 | 4.34E-02 | -0.1749 | down FDR |
| NAAA_Neurology | -0.2536 | 0.4989 | -3.5031 | 5.16E-04 | 4.52E-02 | -0.2982 | down FDR |
| SMPDL3A_Inflammation | -0.2671 | 0.2524 | -3.4780 | 5.65E-04 | 4.77E-02 | -0.3768 | down FDR |

**Table S3.** DTG + 3TC protein levels compared to DTG + TDF/TAF + FTC

Comparing 2DR: DTG+3TC to 3DR: DTG+TDF/TAF+FTC, we observed identified 377 nominally significant proteins, with 42 downregulated (log2FC:-0.8 to -0.1) and one upregulated (CLEC4A, log2FC: 0.2) DEPs in 2DR after FDR adjustment (FDR<0.05)

| **Protein**  **(and Olink® Panel)** | **log FC** | **Average Expression** | **t-value** | **P-value** | **FDR** | **B-value** | **Regulation** |
| --- | --- | --- | --- | --- | --- | --- | --- |
| FABP1_Inflammation | -0.8242 | 1.2223 | -6.4094 | 5.60E-10 | 1.19E-06 | 12.3125 | down FDR |
| RBP2_Oncology | -0.6278 | 1.3299 | -6.3070 | 1.01E-09 | 1.19E-06 | 11.7610 | down FDR |
| FABP2_Cardiometabolic | -0.6471 | 0.9130 | -6.0511 | 4.25E-09 | 2.72E-06 | 10.4122 | down FDR |
| LGALS4_Inflammation | -0.4798 | 0.9099 | -6.0367 | 4.60E-09 | 2.72E-06 | 10.3375 | down FDR |
| ACY3_Cardiometabolic_II | -0.5241 | -0.1686 | -5.8095 | 1.59E-08 | 7.52E-06 | 9.1788 | down FDR |
| GDF15_Cardiometabolic | -0.3983 | 0.6618 | -5.6395 | 3.92E-08 | 1.55E-05 | 8.3350 | down FDR |
| CCL11_Inflammation | -0.2797 | 0.2750 | -5.5748 | 5.50E-08 | 1.86E-05 | 8.0188 | down FDR |
| CKMT1A_CKMT1B_Inflammation | -0.7064 | 1.8830 | -5.4125 | 1.27E-07 | 3.75E-05 | 7.2394 | down FDR |
| EPS8L2_Oncology | -0.2426 | 0.4869 | -5.2761 | 2.52E-07 | 6.63E-05 | 6.5989 | down FDR |
| PDZK1_Inflammation_II | -0.3718 | -0.0623 | -5.2546 | 2.81E-07 | 6.64E-05 | 6.4989 | down FDR |
| DDC_Cardiometabolic | -0.4361 | 0.7509 | -5.1951 | 3.77E-07 | 8.11E-05 | 6.2248 | down FDR |
| DKK4_Neurology | -0.3802 | 1.8324 | -5.0092 | 9.31E-07 | 1.84E-04 | 5.3845 | down FDR |
| CCL25_Inflammation | -0.3664 | 1.0901 | -4.9446 | 1.27E-06 | 2.31E-04 | 5.0984 | down FDR |
| CXCL8_Inflammation | -0.4524 | 0.2368 | -4.8236 | 2.24E-06 | 3.78E-04 | 4.5715 | down FDR |
| PPP1R14D_Neurology_II | -0.3965 | 0.8095 | -4.7535 | 3.10E-06 | 4.89E-04 | 4.2711 | down FDR |
| SULT2A1_Inflammation | -0.4369 | 0.7340 | -4.5087 | 9.34E-06 | 1.38E-03 | 3.2521 | down FDR |
| REG4_Inflammation | -0.2962 | 0.8446 | -4.4833 | 1.04E-05 | 1.45E-03 | 3.1489 | down FDR |
| SHD_Cardiometabolic_II | -0.4367 | -0.0711 | -4.4591 | 1.16E-05 | 1.53E-03 | 3.0511 | down FDR |
| RRM2_Oncology | -0.3106 | -0.2086 | -4.4046 | 1.47E-05 | 1.84E-03 | 2.8327 | down FDR |
| CTSS_Neurology | -0.1450 | 0.6805 | -4.3816 | 1.63E-05 | 1.93E-03 | 2.7413 | down FDR |
| ACY1_Cardiometabolic | -0.4200 | -0.2233 | -4.2641 | 2.69E-05 | 3.03E-03 | 2.2809 | down FDR |
| AGR3_Oncology | -0.5495 | -0.0342 | -4.2297 | 3.11E-05 | 3.34E-03 | 2.1480 | down FDR |
| LGMN_Inflammation | -0.2078 | 0.7312 | -4.1902 | 3.66E-05 | 3.77E-03 | 1.9966 | down FDR |
| CXCL11_Neurology | -0.4658 | 2.1848 | -4.1473 | 4.38E-05 | 4.32E-03 | 1.8337 | down FDR |
| KHK_Cardiometabolic_II | -0.3675 | -0.3073 | -3.9392 | 1.02E-04 | 9.62E-03 | 1.0647 | down FDR |
| OCLN_Neurology_II | -0.2461 | 1.0723 | -3.9099 | 1.14E-04 | 1.04E-02 | 0.9595 | down FDR |
| MUC13_Neurology | -0.2973 | 0.6626 | -3.8417 | 1.49E-04 | 1.31E-02 | 0.7167 | down FDR |
| REG1A_Cardiometabolic | -0.2923 | 0.8424 | -3.8220 | 1.61E-04 | 1.36E-02 | 0.6475 | down FDR |
| NOS2_Cardiometabolic_II | -0.2877 | 0.0011 | -3.7157 | 2.41E-04 | 1.95E-02 | 0.2786 | down FDR |
| HMGCL_Cardiometabolic_II | -0.3010 | -0.1938 | -3.7097 | 2.47E-04 | 1.95E-02 | 0.2583 | down FDR |
| REG1B_Cardiometabolic | -0.2835 | 0.9727 | -3.6363 | 3.25E-04 | 2.48E-02 | 0.0094 | down FDR |
| PMM2_Cardiometabolic_II | -0.3715 | -0.5439 | -3.6184 | 3.47E-04 | 2.57E-02 | -0.0506 | down FDR |
| CLEC4A_Inflammation | 0.2201 | 0.4393 | 3.5955 | 3.78E-04 | 2.71E-02 | -0.1267 | up FDR |
| SCPEP1_Cardiometabolic_II | -0.2412 | -0.1650 | -3.5574 | 4.35E-04 | 3.03E-02 | -0.2527 | down FDR |
| LGALS9_Inflammation | -0.1840 | 0.4790 | -3.5281 | 4.84E-04 | 3.20E-02 | -0.3489 | down FDR |
| IFNL1_Neurology | -0.4889 | 0.1116 | -3.5267 | 4.86E-04 | 3.20E-02 | -0.3533 | down FDR |
| CCL8_Oncology | -0.2688 | -3.1369 | -3.4652 | 6.06E-04 | 3.88E-02 | -0.5524 | down FDR |
| PLXNB2_Cardiometabolic | -0.1324 | 0.6480 | -3.4389 | 6.66E-04 | 4.13E-02 | -0.6366 | down FDR |
| CDH17_Cardiometabolic | -0.3353 | 0.6342 | -3.4331 | 6.80E-04 | 4.13E-02 | -0.6554 | down FDR |
| GYS1_Cardiometabolic | -0.5135 | 4.2623 | -3.4145 | 7.26E-04 | 4.30E-02 | -0.7142 | down FDR |
| VEGFA_Inflammation | -0.2302 | 1.3080 | -3.4002 | 7.64E-04 | 4.41E-02 | -0.7595 | down FDR |
| SYNGAP1_Neurology_II | -0.2947 | -0.6105 | -3.3883 | 7.97E-04 | 4.49E-02 | -0.7971 | down FDR |
| PBLD_Neurology | -0.2661 | 0.0312 | -3.3571 | 8.88E-04 | 4.89E-02 | -0.8947 | down FDR |

**Table S4.** DTG + 3TC protein levels compared to DTG + TDF + FTC

The 2DR: DTG+3TC compared with 3DR: DTG+TDF+FTC showed identified 309 nominally significant proteins, with 63 downregulated (log2FC:-1.4 to -0.1) and two upregulated (Cathepsin E (CTSE) with log2FC:0.5, and Carboxypeptidase A4 (CPA4) with log2FC:0.2) DEPs in 2DR after FDR adjustment.

| **Protein**  **(and Olink® Panel)** | **log FC** | **Average Expression** | **t-value** | **P-value** | **FDR** | **B-value** | **Regulation** |
| --- | --- | --- | --- | --- | --- | --- | --- |
| RBP2_Oncology | -1.1783 | 1.3637 | -10.9127 | 6.41E-23 | 1.52E-19 | 41.1569 | down FDR |
| CKMT1A_CKMT1B_Inflammation | -1.4358 | 1.9584 | -9.9089 | 9.94E-20 | 1.18E-16 | 34.0457 | down FDR |
| FABP2_Cardiometabolic | -1.1900 | 0.9439 | -9.7984 | 2.19E-19 | 1.73E-16 | 33.2796 | down FDR |
| ACY3_Cardiometabolic_II | -0.9502 | -0.1417 | -9.0329 | 4.73E-17 | 2.80E-14 | 28.0807 | down FDR |
| EPS8L2_Oncology | -0.4511 | 0.5018 | -8.7406 | 3.48E-16 | 1.38E-13 | 26.1492 | down FDR |
| DDC_Cardiometabolic | -0.8399 | 0.7866 | -8.7401 | 3.49E-16 | 1.38E-13 | 26.1460 | down FDR |
| LGALS4_Inflammation | -0.7787 | 0.9058 | -8.3444 | 4.94E-15 | 1.67E-12 | 23.5854 | down FDR |
| FABP1_Inflammation | -1.2918 | 1.2085 | -8.3014 | 6.57E-15 | 1.94E-12 | 23.3109 | down FDR |
| PPP1R14D_Neurology_II | -0.7497 | 0.8287 | -7.5791 | 6.84E-13 | 1.80E-10 | 18.8259 | down FDR |
| DKK4_Neurology | -0.6168 | 1.8300 | -6.6006 | 2.45E-10 | 5.79E-08 | 13.1658 | down FDR |
| GDF15_Cardiometabolic | -0.5085 | 0.6256 | -6.3935 | 7.93E-10 | 1.71E-07 | 12.0362 | down FDR |
| IFNL1_Neurology | -0.9797 | 0.1594 | -6.2566 | 1.70E-09 | 3.36E-07 | 11.3037 | down FDR |
| CCL25_Inflammation | -0.5523 | 1.0749 | -6.0904 | 4.24E-09 | 7.72E-07 | 10.4294 | down FDR |
| CTSE_Inflammation_II | 0.5337 | 0.1428 | 5.8807 | 1.31E-08 | 2.21E-06 | 9.3517 | up FDR |
| AGR3_Oncology | -0.8955 | -0.0360 | -5.6234 | 5.01E-08 | 7.91E-06 | 8.0685 | down FDR |
| NOS2_Cardiometabolic_II | -0.5224 | 0.0147 | -5.5605 | 6.91E-08 | 1.02E-05 | 7.7613 | down FDR |
| SULT2A1_Inflammation | -0.6213 | 0.7255 | -5.5035 | 9.23E-08 | 1.22E-05 | 7.4855 | down FDR |
| PDZK1_Inflammation_II | -0.4545 | -0.0890 | -5.5025 | 9.28E-08 | 1.22E-05 | 7.4803 | down FDR |
| CCL11_Inflammation | -0.3356 | 0.2450 | -5.3294 | 2.21E-07 | 2.75E-05 | 6.6561 | down FDR |
| PEPD_Inflammation_II | -0.2723 | -0.0795 | -5.2991 | 2.56E-07 | 3.03E-05 | 6.5143 | down FDR |
| LGMN_Inflammation | -0.3120 | 0.7285 | -5.1942 | 4.28E-07 | 4.82E-05 | 6.0268 | down FDR |
| REG4_Inflammation | -0.4122 | 0.8159 | -5.1769 | 4.65E-07 | 5.00E-05 | 5.9475 | down FDR |
| CDH17_Cardiometabolic | -0.6066 | 0.6502 | -5.0979 | 6.80E-07 | 7.00E-05 | 5.5866 | down FDR |
| OCLN_Neurology_II | -0.3691 | 1.0633 | -5.0162 | 1.00E-06 | 9.88E-05 | 5.2184 | down FDR |
| CXCL11_Neurology | -0.6693 | 2.1720 | -4.9003 | 1.72E-06 | 1.63E-04 | 4.7041 | down FDR |
| EPPK1_Cardiometabolic_II | -0.4491 | -0.1776 | -4.8326 | 2.36E-06 | 2.15E-04 | 4.4083 | down FDR |
| REG1A_Cardiometabolic | -0.4152 | 0.8209 | -4.8172 | 2.53E-06 | 2.22E-04 | 4.3418 | down FDR |
| CTSS_Neurology | -0.1832 | 0.6722 | -4.4975 | 1.05E-05 | 8.92E-04 | 2.9949 | down FDR |
| TPK1_Cardiometabolic_II | -0.2504 | -0.0992 | -4.4255 | 1.44E-05 | 1.17E-03 | 2.7026 | down FDR |
| CLIC5_Neurology_II | -0.2565 | -0.0593 | -4.2831 | 2.63E-05 | 2.08E-03 | 2.1359 | down FDR |
| RRM2_Oncology | -0.3695 | -0.2250 | -4.1950 | 3.80E-05 | 2.90E-03 | 1.7932 | down FDR |
| ACY1_Cardiometabolic | -0.4971 | -0.2605 | -4.1813 | 4.02E-05 | 2.97E-03 | 1.7405 | down FDR |
| KHK_Cardiometabolic_II | -0.4635 | -0.3379 | -4.1314 | 4.92E-05 | 3.53E-03 | 1.5498 | down FDR |
| MUC13_Neurology | -0.3887 | 0.6430 | -4.0801 | 6.06E-05 | 4.22E-03 | 1.3558 | down FDR |
| DCTPP1_Cardiometabolic | -0.2421 | 0.5819 | -3.9421 | 1.05E-04 | 6.80E-03 | 0.8443 | down FDR |
| SPINK4_Inflammation | -0.5010 | 1.7045 | -3.9404 | 1.06E-04 | 6.80E-03 | 0.8382 | down FDR |
| CCL8_Oncology | -0.3737 | -3.1606 | -3.9391 | 1.06E-04 | 6.80E-03 | 0.8332 | down FDR |
| REG1B_Cardiometabolic | -0.3417 | 0.9391 | -3.9235 | 1.13E-04 | 7.00E-03 | 0.7763 | down FDR |
| COL1A1_Cardiometabolic | -0.2982 | 0.3389 | -3.9181 | 1.15E-04 | 7.00E-03 | 0.7568 | down FDR |
| PMM2_Cardiometabolic_II | -0.4882 | -0.5591 | -3.9107 | 1.19E-04 | 7.03E-03 | 0.7299 | down FDR |
| PLA2G10_Neurology | -0.3660 | 0.4799 | -3.8869 | 1.30E-04 | 7.52E-03 | 0.6441 | down FDR |
| MDK_Oncology | -0.2891 | 1.4565 | -3.8496 | 1.50E-04 | 8.48E-03 | 0.5102 | down FDR |
| SHD_Cardiometabolic_II | -0.4296 | -0.1240 | -3.8426 | 1.55E-04 | 8.50E-03 | 0.4852 | down FDR |
| CCL20_Inflammation | -0.5715 | 0.1567 | -3.8174 | 1.70E-04 | 9.16E-03 | 0.3955 | down FDR |
| ATG16L1_Oncology_II | -0.4495 | 1.1611 | -3.7321 | 2.35E-04 | 1.24E-02 | 0.0956 | down FDR |
| FABP6_Cardiometabolic | -0.3922 | 0.8371 | -3.6719 | 2.95E-04 | 1.52E-02 | -0.1119 | down FDR |
| CHAD_Inflammation_II | -0.3223 | 0.0909 | -3.6472 | 3.23E-04 | 1.63E-02 | -0.1964 | down FDR |
| SCPEP1_Cardiometabolic_II | -0.3126 | -0.1802 | -3.6105 | 3.69E-04 | 1.82E-02 | -0.3209 | down FDR |
| IGLON5_Oncology_II | -0.5520 | -0.3150 | -3.5599 | 4.44E-04 | 2.15E-02 | -0.4908 | down FDR |
| SYNGAP1_Neurology_II | -0.3727 | -0.6214 | -3.5182 | 5.16E-04 | 2.44E-02 | -0.6289 | down FDR |
| AREG_Oncology | -0.2394 | 0.7538 | -3.4990 | 5.53E-04 | 2.57E-02 | -0.6922 | down FDR |
| PBLD_Neurology | -0.3360 | 0.0127 | -3.4801 | 5.91E-04 | 2.67E-02 | -0.7538 | down FDR |
| HMGCL_Cardiometabolic_II | -0.3259 | -0.2243 | -3.4766 | 5.99E-04 | 2.67E-02 | -0.7654 | down FDR |
| SPP1_Cardiometabolic | -0.3303 | 0.4173 | -3.4577 | 6.40E-04 | 2.81E-02 | -0.8268 | down FDR |
| CPA4_Inflammation_II | 0.2351 | -0.0181 | 3.4498 | 6.59E-04 | 2.83E-02 | -0.8526 | up FDR |
| SAFB2_Neurology_II | -0.1851 | 0.1691 | -3.3965 | 7.94E-04 | 3.36E-02 | -1.0240 | down FDR |
| LAG3_Oncology | -0.2342 | 0.6500 | -3.3738 | 8.59E-04 | 3.57E-02 | -1.0962 | down FDR |
| MOCS2_Inflammation_II | -0.2989 | -0.2261 | -3.3658 | 8.84E-04 | 3.59E-02 | -1.1217 | down FDR |
| NT5C_Cardiometabolic_II | -0.3572 | -0.2494 | -3.3623 | 8.94E-04 | 3.59E-02 | -1.1327 | down FDR |
| KRT19_Inflammation | -0.4448 | 0.8039 | -3.3363 | 9.78E-04 | 3.80E-02 | -1.2146 | down FDR |
| GSR_Inflammation_II | -0.1318 | -0.0592 | -3.3321 | 9.93E-04 | 3.80E-02 | -1.2278 | down FDR |
| VEGFA_Inflammation | -0.2833 | 1.2848 | -3.3313 | 9.95E-04 | 3.80E-02 | -1.2303 | down FDR |
| PIGR_Neurology | -0.2246 | 0.2488 | -3.3058 | 1.09E-03 | 4.08E-02 | -1.3102 | down FDR |
| P4HB_Oncology | -0.2087 | 0.7674 | -3.2879 | 1.15E-03 | 4.27E-02 | -1.3660 | down FDR |
| ABRAXAS2_Cardiometabolic_II | -0.3518 | -0.4681 | -3.2566 | 1.28E-03 | 4.68E-02 | -1.4626 | down FDR |

**Table S5.** DTG + 3TC protein levels compared to DTG + TAF + FTC

The comparison with 3DR: DTG+TAF+FTC revealed 307 nominally significant proteins, with only one downregulated DEP: C-X-C Motif Chemokine Ligand 8 (CXCL8, log2FC:-0.6) after FDR adjustment.

| **Protein**  **(and Olink® Panel)** | **log FC** | **Average Expression** | **t-value** | **P-value** | **FDR** | **B-value** | **Regulation** |
| --- | --- | --- | --- | --- | --- | --- | --- |
| CXCL8_Inflammation | -0.6051 | 0.2266 | -5.2155 | 3.95E-07 | 9.36E-04 | 5.6798 | down FDR |

1. **Differentially Expressed Genes**

The differentially expressed genes (DEG) were defined as genes with a fold change absolute values greater than 1.5 (to mitigate false positives) and FDR corrected significance (<0.05).

**Table S6.** DTG + 3TC gene levels compared to DTG + 3TC + ABC

| **Gene ID** | **Gene Symbol** | **Gene Type** | **Description** | **Regulation** | **log FC** | **P-value** | **FDR** |
| --- | --- | --- | --- | --- | --- | --- | --- |
| ENSG00000168298.6 | HIST1H1E | protein_coding | H1.4 linker histone, cluster member [Source:HGNC Symbol;Acc:HGNC:4718] | down | -1.55254 | 6.71E-20 | 9.42E-16 |
| ENSG00000125538.11 | IL1B | protein_coding | interleukin 1 beta [Source:HGNC Symbol;Acc:HGNC:5992] | down | -1.35515 | 5.29E-16 | 3.71E-12 |
| ENSG00000204388.6 | HSPA1B | protein_coding | heat shock protein family A (Hsp70) member 1B [Source:HGNC Symbol;Acc:HGNC:5233] | down | -1.25594 | 1.34E-15 | 6.27E-12 |
| ENSG00000143878.9 | RHOB | protein_coding | ras homolog family member B [Source:HGNC Symbol;Acc:HGNC:668] | down | -0.92838 | 2.42E-15 | 8.50E-12 |
| ENSG00000177606.6 | JUN | protein_coding | Jun proto-oncogene, AP-1 transcription factor subunit [Source:HGNC Symbol;Acc:HGNC:6204] | down | -0.66190 | 3.71E-15 | 1.04E-11 |
| ENSG00000120738.7 | EGR1 | protein_coding | early growth response 1 [Source:HGNC Symbol;Acc:HGNC:3238] | down | -1.40889 | 2.81E-14 | 6.57E-11 |
| ENSG00000187837.3 | HIST1H1C | protein_coding | H1.2 linker histone, cluster member [Source:HGNC Symbol;Acc:HGNC:4716] | down | -0.85164 | 3.62E-14 | 7.25E-11 |
| ENSG00000273338.1 | AC103591.3 | antisense_RNA | novel transcript, antisense to DNAJB4 | down | -0.66483 | 8.35E-14 | 1.46E-10 |
| ENSG00000158373.8 | HIST1H2BD | protein_coding | H2B clustered histone 5 [Source:HGNC Symbol;Acc:HGNC:4747] | down | -0.77686 | 4.47E-13 | 6.96E-10 |
| ENSG00000123358.19 | NR4A1 | protein_coding | nuclear receptor subfamily 4 group A member 1 [Source:HGNC Symbol;Acc:HGNC:7980] | down | -0.70450 | 1.31E-12 | 1.84E-09 |
| ENSG00000206828.1 | U1 | snRNA | RNA, variant U1 small nuclear 30 [Source:HGNC Symbol;Acc:HGNC:54434] | down | -0.71409 | 2.99E-12 | 3.81E-09 |
| ENSG00000273802.2 | HIST1H2BG | protein_coding | H2B clustered histone 8 [Source:HGNC Symbol;Acc:HGNC:4746] | down | -0.76430 | 6.36E-12 | 7.43E-09 |
| ENSG00000277075.2 | HIST1H2AE | protein_coding | H2A clustered histone 8 [Source:HGNC Symbol;Acc:HGNC:4724] | down | -0.73345 | 2.03E-11 | 2.19E-08 |
| ENSG00000162772.16 | ATF3 | protein_coding | activating transcription factor 3 [Source:HGNC Symbol;Acc:HGNC:785] | down | -0.65185 | 4.33E-11 | 4.34E-08 |
| ENSG00000270640.1 | AC104695.3 | sense_intronic | novel transcript, sense intronic to FOSL2 | down | -0.63332 | 5.85E-11 | 5.47E-08 |
| ENSG00000274210.1 | U1 | snRNA | RNA, variant U1 small nuclear 27 [Source:HGNC Symbol;Acc:HGNC:54431] | down | -0.74318 | 1.03E-10 | 8.53E-08 |
| ENSG00000216490.3 | IFI30 | protein_coding | IFI30 lysosomal thiol reductase [Source:HGNC Symbol;Acc:HGNC:5398] | down | -0.59897 | 2.39E-09 | 1.46E-06 |
| ENSG00000184260.5 | HIST2H2AC | protein_coding | H2A clustered histone 20 [Source:HGNC Symbol;Acc:HGNC:4738] | down | -0.61164 | 4.82E-09 | 2.43E-06 |
| ENSG00000203814.6 | HIST2H2BF | protein_coding | H2B clustered histone 18 [Source:HGNC Symbol;Acc:HGNC:24700] | down | -0.87091 | 4.85E-09 | 2.43E-06 |
| ENSG00000275538.1 | RNVU1-19 | snRNA | RNA, variant U1 small nuclear 19 [Source:HGNC Symbol;Acc:HGNC:48324] | down | -0.86663 | 7.75E-09 | 3.62E-06 |
| ENSG00000125740.13 | FOSB | protein_coding | FosB proto-oncogene, AP-1 transcription factor subunit [Source:HGNC Symbol;Acc:HGNC:3797] | down | -0.61794 | 6.04E-08 | 1.88E-05 |
| ENSG00000277632.1 | CCL3 | protein_coding | C-C motif chemokine ligand 3 [Source:HGNC Symbol;Acc:HGNC:10627] | down | -0.72230 | 1.95E-07 | 4.03E-05 |
| ENSG00000211896.7 | IGHG1 | IG_C_gene | immunoglobulin heavy constant gamma 1 (G1m marker) [Source:HGNC Symbol;Acc:HGNC:5525] | down | -0.62839 | 2.04E-07 | 4.09E-05 |
| ENSG00000169429.10 | CXCL8 | protein_coding | C-X-C motif chemokine ligand 8 [Source:HGNC Symbol;Acc:HGNC:6025] | down | -0.75698 | 1.24E-06 | 1.75E-04 |
| ENSG00000276085.1 | CCL3L3 | protein_coding | C-C motif chemokine ligand 3 like 3 [Source:HGNC Symbol;Acc:HGNC:30554] | down | -0.76255 | 2.51E-06 | 3.01E-04 |

**Table S7.** DTG + 3TC gene levels compared to DTG + TDF/TAF + FTC

| **Gene ID** | **Gene Symbol** | **Gene Type** | **Description** | **Regulation** | **log FC** | **P-value** | **FDR** |
| --- | --- | --- | --- | --- | --- | --- | --- |
| ENSG00000175606.10 | TMEM70 | protein_coding | transmembrane protein 70 [Source:HGNC Symbol;Acc:HGNC:26050] | down | -0.6134 | 2.57E-21 | 3.91E-17 |
| ENSG00000168298.6 | HIST1H1E | protein_coding | H1.4 linker histone, cluster member [Source:HGNC Symbol;Acc:HGNC:4718] | down | -1.6357 | 1.96E-17 | 1.49E-13 |
| ENSG00000124575.6 | HIST1H1D | protein_coding | H1.3 linker histone, cluster member [Source:HGNC Symbol;Acc:HGNC:4717] | down | -1.3424 | 3.16E-13 | 1.20E-09 |
| ENSG00000187837.3 | HIST1H1C | protein_coding | H1.2 linker histone, cluster member [Source:HGNC Symbol;Acc:HGNC:4716] | down | -0.8422 | 1.76E-12 | 5.33E-09 |
| ENSG00000204388.6 | HSPA1B | protein_coding | heat shock protein family A (Hsp70) member 1B [Source:HGNC Symbol;Acc:HGNC:5233] | down | -1.2030 | 5.66E-12 | 1.43E-08 |
| ENSG00000143878.9 | RHOB | protein_coding | ras homolog family member B [Source:HGNC Symbol;Acc:HGNC:668] | down | -0.8501 | 2.04E-11 | 3.73E-08 |
| ENSG00000277075.2 | HIST1H2AE | protein_coding | H2A clustered histone 8 [Source:HGNC Symbol;Acc:HGNC:4724] | down | -0.8163 | 3.53E-11 | 5.36E-08 |
| ENSG00000260401.1 | AP002761.4 | sense_overlapping | novel transcript, overlapping to P2RY2 | up | 0.6037 | 9.37E-11 | 1.29E-07 |
| ENSG00000120738.7 | EGR1 | protein_coding | early growth response 1 [Source:HGNC Symbol;Acc:HGNC:3238] | down | -1.2810 | 1.73E-10 | 2.02E-07 |
| ENSG00000158373.8 | HIST1H2BD | protein_coding | H2B clustered histone 5 [Source:HGNC Symbol;Acc:HGNC:4747] | down | -0.7209 | 2.14E-10 | 2.33E-07 |
| ENSG00000273802.2 | HIST1H2BG | protein_coding | H2B clustered histone 8 [Source:HGNC Symbol;Acc:HGNC:4746] | down | -0.7596 | 2.73E-10 | 2.59E-07 |
| ENSG00000203814.6 | HIST2H2BF | protein_coding | H2B clustered histone 18 [Source:HGNC Symbol;Acc:HGNC:24700] | down | -1.0503 | 4.63E-10 | 3.71E-07 |
| ENSG00000123689.5 | G0S2 | protein_coding | G0/G1 switch 2 [Source:HGNC Symbol;Acc:HGNC:30229] | down | -0.9223 | 2.04E-09 | 1.35E-06 |
| ENSG00000169429.10 | CXCL8 | protein_coding | C-X-C motif chemokine ligand 8 [Source:HGNC Symbol;Acc:HGNC:6025] | down | -1.0587 | 2.80E-09 | 1.64E-06 |
| ENSG00000184260.5 | HIST2H2AC | protein_coding | H2A clustered histone 20 [Source:HGNC Symbol;Acc:HGNC:4738] | down | -0.6828 | 2.97E-09 | 1.67E-06 |
| ENSG00000206828.1 | U1 | snRNA | RNA, variant U1 small nuclear 30 [Source:HGNC Symbol;Acc:HGNC:54434] | down | -0.6304 | 2.46E-08 | 7.05E-06 |
| ENSG00000168329.13 | CX3CR1 | protein_coding | C-X3-C motif chemokine receptor 1 [Source:HGNC Symbol;Acc:HGNC:2558] | down | -0.6350 | 3.47E-08 | 8.51E-06 |
| ENSG00000277632.1 | CCL3 | protein_coding | C-C motif chemokine ligand 3 [Source:HGNC Symbol;Acc:HGNC:10627] | down | -0.8201 | 7.45E-08 | 1.51E-05 |
| ENSG00000109814.11 | UGDH | protein_coding | UDP-glucose 6-dehydrogenase [Source:HGNC Symbol;Acc:HGNC:12525] | down | -0.5947 | 4.84E-07 | 4.58E-05 |
| ENSG00000125538.11 | IL1B | protein_coding | interleukin 1 beta [Source:HGNC Symbol;Acc:HGNC:5992] | down | -0.8346 | 7.16E-07 | 5.91E-05 |
| ENSG00000182253.14 | SYNM | protein_coding | synemin [Source:HGNC Symbol;Acc:HGNC:24466] | up | 0.5999 | 7.69E-07 | 6.15E-05 |
| ENSG00000255398.2 | HCAR3 | protein_coding | hydroxycarboxylic acid receptor 3 [Source:HGNC Symbol;Acc:HGNC:16824] | down | -0.6671 | 7.81E-07 | 6.18E-05 |
| ENSG00000137834.14 | SMAD6 | protein_coding | SMAD family member 6 [Source:HGNC Symbol;Acc:HGNC:6772] | up | 0.7013 | 9.46E-07 | 7.12E-05 |
| ENSG00000181649.5 | PHLDA2 | protein_coding | pleckstrin homology like domain family A member 2 [Source:HGNC Symbol;Acc:HGNC:12385] | down | -0.6032 | 1.41E-06 | 8.66E-05 |
| ENSG00000073756.11 | PTGS2 | protein_coding | prostaglandin-endoperoxide synthase 2 [Source:HGNC Symbol;Acc:HGNC:9605] | down | -0.7687 | 2.37E-06 | 1.20E-04 |
| ENSG00000276085.1 | CCL3L3 | protein_coding | C-C motif chemokine ligand 3 like 3 [Source:HGNC Symbol;Acc:HGNC:30554] | down | -0.8238 | 3.02E-06 | 1.41E-04 |
| ENSG00000275538.1 | RNVU1-19 | snRNA | RNA, variant U1 small nuclear 19 [Source:HGNC Symbol;Acc:HGNC:48324] | down | -0.7855 | 5.42E-06 | 1.98E-04 |
| ENSG00000125740.13 | FOSB | protein_coding | FosB proto-oncogene, AP-1 transcription factor subunit [Source:HGNC Symbol;Acc:HGNC:3797] | down | -0.5927 | 6.68E-06 | 2.22E-04 |
| ENSG00000163739.4 | CXCL1 | protein_coding | C-X-C motif chemokine ligand 1 [Source:HGNC Symbol;Acc:HGNC:4602] | down | -0.6822 | 4.91E-05 | 7.29E-04 |
| ENSG00000275063.1 | AC233755.1 | protein_coding |  | down | -0.6531 | 1.90E-04 | 1.78E-03 |
| ENSG00000148344.10 | PTGES | protein_coding | prostaglandin E synthase [Source:HGNC Symbol;Acc:HGNC:9599] | up | 0.5961 | 2.11E-04 | 1.91E-03 |

**Table S8.** DTG + 3TC gene levels compared to DTG + TDF + FTC

| **Gene ID** | **Gene Symbol** | **Gene Type** | **Description** | **Regulation** | **log FC** | **P-value** | **FDR** |
| --- | --- | --- | --- | --- | --- | --- | --- |
| ENSG00000100721.10 | TCL1A | protein_coding | TCL1 family AKT coactivator A [Source:HGNC Symbol;Acc:HGNC:11648] | down | -0.6575 | 4.72E-05 | 9.01E-03 |
| ENSG00000120738.7 | EGR1 | protein_coding | early growth response 1 [Source:HGNC Symbol;Acc:HGNC:3238] | down | -0.9227 | 4.51E-05 | 8.94E-03 |
| ENSG00000123689.5 | G0S2 | protein_coding | G0/G1 switch 2 [Source:HGNC Symbol;Acc:HGNC:30229] | down | -0.7387 | 2.24E-05 | 6.54E-03 |
| ENSG00000124575.6 | HIST1H1D | protein_coding | H1.3 linker histone, cluster member [Source:HGNC Symbol;Acc:HGNC:4717] | down | -0.9745 | 2.69E-06 | 2.19E-03 |
| ENSG00000125538.11 | IL1B | protein_coding | interleukin 1 beta [Source:HGNC Symbol;Acc:HGNC:5992] | down | -0.6808 | 2.58E-04 | 1.59E-02 |
| ENSG00000128218.7 | VPREB3 | protein_coding | V-set pre-B cell surrogate light chain 3 [Source:HGNC Symbol;Acc:HGNC:12710] | down | -0.5869 | 3.44E-05 | 8.04E-03 |
| ENSG00000132185.16 | FCRLA | protein_coding | Fc receptor like A [Source:HGNC Symbol;Acc:HGNC:18504] | down | -0.6837 | 8.30E-05 | 1.14E-02 |
| ENSG00000143878.9 | RHOB | protein_coding | ras homolog family member B [Source:HGNC Symbol;Acc:HGNC:668] | down | -0.7335 | 5.97E-07 | 1.25E-03 |
| ENSG00000158373.8 | HIST1H2BD | protein_coding | H2B clustered histone 5 [Source:HGNC Symbol;Acc:HGNC:4747] | down | -0.5859 | 7.11E-06 | 3.89E-03 |
| ENSG00000168298.6 | HIST1H1E | protein_coding | H1.4 linker histone, cluster member [Source:HGNC Symbol;Acc:HGNC:4718] | down | -1.2203 | 2.85E-08 | 1.49E-04 |
| ENSG00000169429.10 | CXCL8 | protein_coding | C-X-C motif chemokine ligand 8 [Source:HGNC Symbol;Acc:HGNC:6025] | down | -0.7850 | 9.92E-05 | 1.17E-02 |
| ENSG00000172264.16 | MACROD2 | protein_coding | mono-ADP ribosylhydrolase 2 [Source:HGNC Symbol;Acc:HGNC:16126] | down | -0.6486 | 2.11E-05 | 6.34E-03 |
| ENSG00000187837.3 | HIST1H1C | protein_coding | H1.2 linker histone, cluster member [Source:HGNC Symbol;Acc:HGNC:4716] | down | -0.6464 | 1.75E-06 | 1.82E-03 |
| ENSG00000203814.6 | HIST2H2BF | protein_coding | H2B clustered histone 18 [Source:HGNC Symbol;Acc:HGNC:24700] | down | -0.8179 | 1.95E-05 | 6.10E-03 |
| ENSG00000204388.6 | HSPA1B | protein_coding | heat shock protein family A (Hsp70) member 1B [Source:HGNC Symbol;Acc:HGNC:5233] | down | -0.8057 | 2.47E-05 | 6.67E-03 |
| ENSG00000211896.7 | IGHG1 | IG_C_gene | immunoglobulin heavy constant gamma 1 (G1m marker) [Source:HGNC Symbol;Acc:HGNC:5525] | down | -0.7160 | 2.56E-06 | 2.19E-03 |
| ENSG00000211956.2 | IGHV4-34 | IG_V_gene | immunoglobulin heavy variable 4-34 [Source:HGNC Symbol;Acc:HGNC:5650] | down | -0.6382 | 1.11E-06 | 1.67E-03 |
| ENSG00000253755.1 | IGHGP | IG_C_pseudogene | immunoglobulin heavy constant gamma P (non-functional) [Source:HGNC Symbol;Acc:HGNC:5529] | down | -0.8480 | 1.12E-04 | 1.21E-02 |
| ENSG00000257275.6 | AL139020.1 | antisense_RNA | novel transcript, antisense to TCL1A | down | -0.7650 | 1.20E-04 | 1.24E-02 |
| ENSG00000275538.1 | RNVU1-19 | snRNA | RNA, variant U1 small nuclear 19 [Source:HGNC Symbol;Acc:HGNC:48324] | down | -0.5978 | 1.03E-03 | 2.64E-02 |
| ENSG00000276566.1 | IGKV1D-13 | IG_V_gene | immunoglobulin kappa variable 1D-13 [Source:HGNC Symbol;Acc:HGNC:5747] | down | -1.7107 | 7.21E-07 | 1.25E-03 |
| ENSG00000277075.2 | HIST1H2AE | protein_coding | H2A clustered histone 8 [Source:HGNC Symbol;Acc:HGNC:4724] | down | -0.6256 | 7.21E-06 | 3.89E-03 |
| ENSG00000277224.2 | HIST1H2BF | protein_coding | H2B clustered histone 7 [Source:HGNC Symbol;Acc:HGNC:4752] | down | -0.6130 | 8.81E-05 | 1.17E-02 |

**Table S9.** DTG + 3TC gene levels compared to DTG + TAF + FTC

| **Gene ID** | **Gene Symbol** | **Gene Type** | **Description** | **Regulation** | **log FC** | **P-value** | **FDR** |
| --- | --- | --- | --- | --- | --- | --- | --- |
| ENSG00000175606.10 | TMEM70 | protein_coding | transmembrane protein 70 [Source:HGNC Symbol;Acc:HGNC:26050] | down | -0.6544 | 8.41E-18 | 1.36E-13 |
| ENSG00000168298.6 | HIST1H1E | protein_coding | H1.4 linker histone, cluster member [Source:HGNC Symbol;Acc:HGNC:4718] | down | -1.9626 | 4.46E-16 | 3.60E-12 |
| ENSG00000276966.2 | HIST1H4E | protein_coding | H4 clustered histone 5 [Source:HGNC Symbol;Acc:HGNC:4790] | down | -1.6537 | 4.95E-15 | 2.67E-11 |
| ENSG00000124575.6 | HIST1H1D | protein_coding | H1.3 linker histone, cluster member [Source:HGNC Symbol;Acc:HGNC:4717] | down | -1.6260 | 1.98E-12 | 8.02E-09 |
| ENSG00000204388.6 | HSPA1B | protein_coding | heat shock protein family A (Hsp70) member 1B [Source:HGNC Symbol;Acc:HGNC:5233] | down | -1.5069 | 5.32E-12 | 1.72E-08 |
| ENSG00000187837.3 | HIST1H1C | protein_coding | H1.2 linker histone, cluster member [Source:HGNC Symbol;Acc:HGNC:4716] | down | -1.0081 | 2.63E-11 | 6.56E-08 |
| ENSG00000277075.2 | HIST1H2AE | protein_coding | H2A clustered histone 8 [Source:HGNC Symbol;Acc:HGNC:4724] | down | -0.9699 | 7.54E-11 | 1.53E-07 |
| ENSG00000273802.2 | HIST1H2BG | protein_coding | H2B clustered histone 8 [Source:HGNC Symbol;Acc:HGNC:4746] | down | -0.9043 | 9.87E-10 | 1.77E-06 |
| ENSG00000099860.8 | GADD45B | protein_coding | growth arrest and DNA damage inducible beta [Source:HGNC Symbol;Acc:HGNC:4096] | down | -0.6035 | 1.25E-09 | 1.84E-06 |
| ENSG00000184260.5 | HIST2H2AC | protein_coding | H2A clustered histone 20 [Source:HGNC Symbol;Acc:HGNC:4738] | down | -0.8879 | 1.19E-09 | 1.84E-06 |
| ENSG00000143878.9 | RHOB | protein_coding | ras homolog family member B [Source:HGNC Symbol;Acc:HGNC:668] | down | -0.9304 | 1.55E-09 | 2.10E-06 |
| ENSG00000120738.7 | EGR1 | protein_coding | early growth response 1 [Source:HGNC Symbol;Acc:HGNC:3238] | down | -1.5272 | 2.74E-09 | 3.41E-06 |
| ENSG00000128016.5 | ZFP36 | protein_coding | ZFP36 ring finger protein [Source:HGNC Symbol;Acc:HGNC:12862] | down | -0.6581 | 8.26E-09 | 8.90E-06 |
| ENSG00000158373.8 | HIST1H2BD | protein_coding | H2B clustered histone 5 [Source:HGNC Symbol;Acc:HGNC:4747] | down | -0.8162 | 9.88E-09 | 9.40E-06 |
| ENSG00000277224.2 | HIST1H2BF | protein_coding | H2B clustered histone 7 [Source:HGNC Symbol;Acc:HGNC:4752] | down | -0.9855 | 9.77E-09 | 9.40E-06 |
| ENSG00000203814.6 | HIST2H2BF | protein_coding | H2B clustered histone 18 [Source:HGNC Symbol;Acc:HGNC:24700] | down | -1.2109 | 1.33E-08 | 1.20E-05 |
| ENSG00000168310.10 | IRF2 | protein_coding | interferon regulatory factor 2 [Source:HGNC Symbol;Acc:HGNC:6117] | down | -0.6373 | 2.38E-08 | 2.03E-05 |
| ENSG00000169429.10 | CXCL8 | protein_coding | C-X-C motif chemokine ligand 8 [Source:HGNC Symbol;Acc:HGNC:6025] | down | -1.2681 | 2.71E-08 | 2.03E-05 |
| ENSG00000271913.5 | AL035530.2 | antisense_RNA | TAGAP antisense RNA 1 [Source:HGNC Symbol;Acc:HGNC:55239] | down | -0.6567 | 3.26E-08 | 2.11E-05 |
| ENSG00000277632.1 | CCL3 | protein_coding | C-C motif chemokine ligand 3 [Source:HGNC Symbol;Acc:HGNC:10627] | down | -1.0685 | 3.22E-08 | 2.11E-05 |
| ENSG00000206828.1 | U1 | snRNA | RNA, variant U1 small nuclear 30 [Source:HGNC Symbol;Acc:HGNC:54434] | down | -0.7944 | 3.64E-08 | 2.12E-05 |
| ENSG00000177606.6 | JUN | protein_coding | Jun proto-oncogene, AP-1 transcription factor subunit [Source:HGNC Symbol;Acc:HGNC:6204] | down | -0.6063 | 5.12E-08 | 2.51E-05 |
| ENSG00000088986.10 | DYNLL1 | protein_coding | dynein light chain LC8-type 1 [Source:HGNC Symbol;Acc:HGNC:15476] | down | -0.5855 | 1.13E-07 | 4.16E-05 |
| ENSG00000118515.11 | SGK1 | protein_coding | serum/glucocorticoid regulated kinase 1 [Source:HGNC Symbol;Acc:HGNC:10810] | down | -0.7031 | 1.25E-07 | 4.38E-05 |
| ENSG00000123689.5 | G0S2 | protein_coding | G0/G1 switch 2 [Source:HGNC Symbol;Acc:HGNC:30229] | down | -1.0296 | 1.44E-07 | 4.84E-05 |
| ENSG00000273338.1 | AC103591.3 | antisense_RNA | novel transcript, antisense to DNAJB4 | down | -0.6287 | 2.09E-07 | 6.49E-05 |
| ENSG00000280194.1 | AD000864.1 | TEC | novel transcript | down | -0.7301 | 2.13E-07 | 6.49E-05 |
| ENSG00000136603.13 | SKIL | protein_coding | SKI like proto-oncogene [Source:HGNC Symbol;Acc:HGNC:10897] | up | 0.6690 | 2.29E-07 | 6.73E-05 |
| ENSG00000183978.7 | COA3 | protein_coding | cytochrome c oxidase assembly factor 3 [Source:HGNC Symbol;Acc:HGNC:24990] | down | -0.6579 | 2.40E-07 | 6.80E-05 |
| ENSG00000111181.12 | SLC6A12 | protein_coding | solute carrier family 6 member 12 [Source:HGNC Symbol;Acc:HGNC:11045] | down | -0.7332 | 3.97E-07 | 9.78E-05 |
| ENSG00000260401.1 | AP002761.4 | sense_overlapping | novel transcript, overlapping to P2RY2 | up | 0.5957 | 3.94E-07 | 9.78E-05 |
| ENSG00000137834.14 | SMAD6 | protein_coding | SMAD family member 6 [Source:HGNC Symbol;Acc:HGNC:6772] | up | 0.9072 | 4.20E-07 | 9.86E-05 |
| ENSG00000275791.1 | TRBV10-3 | TR_V_gene | T cell receptor beta variable 10-3 [Source:HGNC Symbol;Acc:HGNC:12179] | down | -0.5965 | 4.47E-07 | 1.01E-04 |
| ENSG00000148344.10 | PTGES | protein_coding | prostaglandin E synthase [Source:HGNC Symbol;Acc:HGNC:9599] | up | 1.1227 | 6.19E-07 | 1.26E-04 |
| ENSG00000174600.13 | CMKLR1 | protein_coding | chemerin chemokine-like receptor 1 [Source:HGNC Symbol;Acc:HGNC:2121] | down | -0.7339 | 7.47E-07 | 1.39E-04 |
| ENSG00000111537.4 | IFNG | protein_coding | interferon gamma [Source:HGNC Symbol;Acc:HGNC:5438] | down | -0.6219 | 9.78E-07 | 1.63E-04 |
| ENSG00000168329.13 | CX3CR1 | protein_coding | C-X3-C motif chemokine receptor 1 [Source:HGNC Symbol;Acc:HGNC:2558] | down | -0.6700 | 1.19E-06 | 1.89E-04 |
| ENSG00000255398.2 | HCAR3 | protein_coding | hydroxycarboxylic acid receptor 3 [Source:HGNC Symbol;Acc:HGNC:16824] | down | -0.8395 | 1.58E-06 | 2.28E-04 |
| ENSG00000229692.3 | SOS1-IT1 | sense_intronic | SOS1 intronic transcript 1 [Source:HGNC Symbol;Acc:HGNC:41385] | down | -0.7099 | 1.98E-06 | 2.57E-04 |
| ENSG00000270640.1 | AC104695.3 | sense_intronic | novel transcript, sense intronic to FOSL2 | down | -0.6053 | 2.06E-06 | 2.65E-04 |
| ENSG00000188886.3 | ASTL | protein_coding | astacin like metalloendopeptidase [Source:HGNC Symbol;Acc:HGNC:31704] | up | 0.7382 | 2.65E-06 | 3.10E-04 |
| ENSG00000181649.5 | PHLDA2 | protein_coding | pleckstrin homology like domain family A member 2 [Source:HGNC Symbol;Acc:HGNC:12385] | down | -0.7418 | 3.81E-06 | 3.91E-04 |
| ENSG00000207205.1 | RNVU1-15 | snRNA | RNA, variant U1 small nuclear 15 [Source:HGNC Symbol;Acc:HGNC:48320] | down | -0.7331 | 4.20E-06 | 4.17E-04 |
| ENSG00000160460.15 | SPTBN4 | protein_coding | spectrin beta, non-erythrocytic 4 [Source:HGNC Symbol;Acc:HGNC:14896] | up | 0.8485 | 4.82E-06 | 4.43E-04 |
| ENSG00000107719.8 | PALD1 | protein_coding | phosphatase domain containing paladin 1 [Source:HGNC Symbol;Acc:HGNC:23530] | up | 0.6741 | 5.21E-06 | 4.60E-04 |
| ENSG00000268355.1 | AC243960.3 | lincRNA | novel transcript | down | -0.6417 | 5.21E-06 | 4.60E-04 |
| ENSG00000215267.8 | AKR1C7P | transcribed_unprocessed_pseudogene | aldo-keto reductase family 1 member C7, pseudogene [Source:HGNC Symbol;Acc:HGNC:44681] | down | -0.5977 | 5.42E-06 | 4.64E-04 |
| ENSG00000127951.6 | FGL2 | protein_coding | fibrinogen like 2 [Source:HGNC Symbol;Acc:HGNC:3696] | down | -0.6200 | 6.27E-06 | 5.00E-04 |
| ENSG00000122877.15 | EGR2 | protein_coding | early growth response 2 [Source:HGNC Symbol;Acc:HGNC:3239] | down | -0.6738 | 6.37E-06 | 5.03E-04 |
| ENSG00000120875.8 | DUSP4 | protein_coding | dual specificity phosphatase 4 [Source:HGNC Symbol;Acc:HGNC:3070] | up | 0.7311 | 6.45E-06 | 5.04E-04 |
| ENSG00000279491.1 | AP003733.4 | TEC | TEC | down | -0.6563 | 6.65E-06 | 5.12E-04 |
| ENSG00000276085.1 | CCL3L3 | protein_coding | C-C motif chemokine ligand 3 like 3 [Source:HGNC Symbol;Acc:HGNC:30554] | down | -1.0121 | 1.47E-05 | 7.88E-04 |
| ENSG00000154319.14 | FAM167A | protein_coding | family with sequence similarity 167 member A [Source:HGNC Symbol;Acc:HGNC:15549] | up | 0.5918 | 1.64E-05 | 8.43E-04 |
| ENSG00000073756.11 | PTGS2 | protein_coding | prostaglandin-endoperoxide synthase 2 [Source:HGNC Symbol;Acc:HGNC:9605] | down | -0.8932 | 1.67E-05 | 8.46E-04 |
| ENSG00000140092.14 | FBLN5 | protein_coding | fibulin 5 [Source:HGNC Symbol;Acc:HGNC:3602] | down | -0.6853 | 2.41E-05 | 1.04E-03 |
| ENSG00000113763.10 | UNC5A | protein_coding | unc-5 netrin receptor A [Source:HGNC Symbol;Acc:HGNC:12567] | down | -0.6157 | 2.54E-05 | 1.08E-03 |
| ENSG00000125538.11 | IL1B | protein_coding | interleukin 1 beta [Source:HGNC Symbol;Acc:HGNC:5992] | down | -0.8850 | 2.54E-05 | 1.08E-03 |
| ENSG00000137507.11 | LRRC32 | protein_coding | leucine rich repeat containing 32 [Source:HGNC Symbol;Acc:HGNC:4161] | up | 0.6615 | 2.69E-05 | 1.11E-03 |
| ENSG00000125740.13 | FOSB | protein_coding | FosB proto-oncogene, AP-1 transcription factor subunit [Source:HGNC Symbol;Acc:HGNC:3797] | down | -0.6949 | 3.95E-05 | 1.35E-03 |
| ENSG00000211804.3 | TRDV1 | TR_V_gene | T cell receptor delta variable 1 [Source:HGNC Symbol;Acc:HGNC:12262] | down | -0.7466 | 3.96E-05 | 1.35E-03 |
| ENSG00000204936.9 | CD177 | protein_coding | CD177 molecule [Source:HGNC Symbol;Acc:HGNC:30072] | down | -1.0132 | 4.04E-05 | 1.36E-03 |
| ENSG00000279602.1 | AC109326.1 | TEC | TEC | down | -0.6484 | 4.10E-05 | 1.36E-03 |
| ENSG00000135636.13 | DYSF | protein_coding | dysferlin [Source:HGNC Symbol;Acc:HGNC:3097] | down | -0.5857 | 4.11E-05 | 1.36E-03 |
| ENSG00000182253.14 | SYNM | protein_coding | synemin [Source:HGNC Symbol;Acc:HGNC:24466] | up | 0.6249 | 4.22E-05 | 1.38E-03 |
| ENSG00000275538.1 | RNVU1-19 | snRNA | RNA, variant U1 small nuclear 19 [Source:HGNC Symbol;Acc:HGNC:48324] | down | -0.8534 | 6.00E-05 | 1.68E-03 |
| ENSG00000274210.1 | U1 | snRNA | RNA, variant U1 small nuclear 27 [Source:HGNC Symbol;Acc:HGNC:54431] | down | -0.5969 | 6.12E-05 | 1.69E-03 |
| ENSG00000145911.5 | N4BP3 | protein_coding | NEDD4 binding protein 3 [Source:HGNC Symbol;Acc:HGNC:29852] | up | 0.5925 | 6.44E-05 | 1.73E-03 |
| ENSG00000170396.7 | ZNF804A | protein_coding | zinc finger protein 804A [Source:HGNC Symbol;Acc:HGNC:21711] | down | -0.6145 | 6.44E-05 | 1.73E-03 |
| ENSG00000173262.11 | SLC2A14 | protein_coding | solute carrier family 2 member 14 [Source:HGNC Symbol;Acc:HGNC:18301] | down | -1.1490 | 8.21E-05 | 2.01E-03 |
| ENSG00000198947.15 | DMD | protein_coding | dystrophin [Source:HGNC Symbol;Acc:HGNC:2928] | up | 0.6583 | 1.25E-04 | 2.55E-03 |
| ENSG00000163739.4 | CXCL1 | protein_coding | C-X-C motif chemokine ligand 1 [Source:HGNC Symbol;Acc:HGNC:4602] | down | -0.7921 | 1.89E-04 | 3.21E-03 |
| ENSG00000261054.1 | AC036108.2 | antisense_RNA | SYNM antisense RNA 2 [Source:HGNC Symbol;Acc:HGNC:56228] | up | 0.7001 | 2.18E-04 | 3.46E-03 |
| ENSG00000101187.15 | SLCO4A1 | protein_coding | solute carrier organic anion transporter family member 4A1 [Source:HGNC Symbol;Acc:HGNC:10953] | up | 0.6400 | 2.52E-04 | 3.78E-03 |
| ENSG00000229436.1 | AC073850.1 | processed_pseudogene | pseudogene similar to part of transcription factor Dp-1 TFDP1 | down | -0.6353 | 2.98E-04 | 4.21E-03 |
| ENSG00000202019.1 | Y_RNA | misc_RNA | Y RNA [Source:RFAM;Acc:RF00019] | down | -0.7384 | 4.07E-04 | 4.98E-03 |
| ENSG00000257743.8 | MGAM2 | protein_coding | maltase-glucoamylase 2 (putative) [Source:HGNC Symbol;Acc:HGNC:28101] | down | -0.7029 | 1.16E-03 | 9.64E-03 |
| ENSG00000211642.3 | IGLV10-54 | IG_V_gene | immunoglobulin lambda variable 10-54 [Source:HGNC Symbol;Acc:HGNC:5884] | down | -0.6455 | 2.07E-03 | 1.41E-02 |
| ENSG00000157551.17 | KCNJ15 | protein_coding | potassium inwardly rectifying channel subfamily J member 15 [Source:HGNC Symbol;Acc:HGNC:6261] | down | -0.6979 | 2.09E-03 | 1.41E-02 |

1. **Pathway Enrichment Analysis - Proteins**

The following table lists the significant enriched pathways identified from the proteomics data, where enrichment analysis was performed on the top 100 most significant proteins, with only those pathways having an FDR-corrected p-value of less than 0.05 and downregulated in the DTG + 3TC group compared to the 3DR group were included.

**Table S10.** Significant pathways (FDR < 0.05) for the proteins in DTG + 3TC compared to DTG + 3TC + ABC

| **Category** | **Term** | **Count** | **P-value** | **Proteins** | **Fold Enrichment** | **FDR** |
| --- | --- | --- | --- | --- | --- | --- |
| KEGG_PATHWAY | hsa01100:Metabolic pathways | 35 | 2.67E-10 | DDC, MOCS2, ECHS1, ASAH2, MVK, ACY1, IDUA, ENO1, KHK, NT5C, HMGCL, GYS1, NT5E, SMPD1, KYNU, CHAC2, ACAA1, GGT1, ATP6V1F, RRM2, PMM2, SORD, SUOX, SDHB, DCTD, GRHPR, FOLH1, VNN1, RRM2B, GSTA3, GSTA1, AGXT, GLA, DCTPP1, GAPDH | 2.8912 | 4.68E-08 |
| REACTOME_PATHWAY | R-HSA-1430728~Metabolism | 37 | 3.17E-08 | DDC, MOCS2, ECHS1, ASAH2, MVK, ACY1, IDUA, ENO1, KHK, NT5C, HMGCL, GYS1, NT5E, SMPD1, KYNU, CHAC2, LDLRAP1, CRYM, ACAA1, GGT1, RRM2, SORD, ACOT13, SUOX, SDHB, DCTD, GRHPR, FOLH1, VNN1, RRM2B, GSTA3, GSTA1, AGXT, GLA, DCTPP1, GAPDH, LGMN | 2.4359 | 1.45E-05 |
| GOTERM_CC_DIRECT | GO:0070062~extracellular exosome | 41 | 6.49E-06 | DDC, ASAH2, ACY1, IDUA, ENO1, PVR, PBLD, KHK, NT5C, PRDX5, NT5E, SMPD1, LAMP2, CHMP1A, EPS8L2, CTSF, CRYM, GGT1, S100A11, ATP6V1F, LYN, MME, ANXA3, F12, SORD, BAIAP2, VAMP8, BST2, ACE2, GRHPR, FOLH1, HGS, GSTA3, NAAA, GSTA1, GPRC5C, GLA, GAPDH, VAMP5, SMPDL3A, LGMN | 1.9094 | 1.19E-03 |
| GOTERM_CC_DIRECT | GO:0005782~peroxisomal matrix | 6 | 3.31E-05 | HMGCL, GRHPR, PRDX5, CRYM, AGXT, ACAA1 | 14.2906 | 3.04E-03 |
| GOTERM_CC_DIRECT | GO:0005829~cytosol | 48 | 1.01E-04 | DDC, MOCS2, MVK, ACY1, ENO1, FUOM, KHK, NT5C, HMGCL, GYS1, PRDX5, NT5E, CASP3, KYNU, CHMP1A, S100A12, EPS8L2, CHAC2, LDLRAP1, CRYM, ACAA1, BID, HGFAC, ATP6V1F, LYN, FIS1, STAT5B, RRM2, MPIG6B, PMM2, SORD, EIF2AK2, ACOT13, BAIAP2, VAMP8, DCTD, GRHPR, EHD3, RRM2B, HGS, GSTA3, GSTA1, HCLS1, SUGT1, AGXT, DCTPP1, GAPDH, SDCCAG8 | 1.5863 | 6.17E-03 |
| GOTERM_CC_DIRECT | GO:0005777~peroxisome | 6 | 5.55E-04 | FIS1, HMGCL, PRDX5, MVK, AGXT, ACAA1 | 8.3362 | 2.55E-02 |

**Table S11.** Significant pathways (FDR < 0.05) for the proteins in DTG + 3TC compared to DTG + TDF/TAF + FTC

| **Category** | **Term** | **Count** | **P-value** | **Proteins** | **Fold Enrichment** | **FDR** |
| --- | --- | --- | --- | --- | --- | --- |
| GOTERM_BP_DIRECT | GO:0061844~antimicrobial humoral immune response mediated by antimicrobial peptide | 12 | 6.66E-07 | CCL25, CXCL11, CXCL9, CCL8, CCL11, CXCL8, CCL7, REG1B, CCL4, REG1A, CCL2, GAPDH | 6.8019 | 5.04E-04 |
| REACTOME_PATHWAY | R-HSA-1430728~Metabolism | 31 | 4.78E-06 | DDC, ACY3, ECHS1, MVK, ACY1, IDUA, GLO1, PTS, KHK, NT5C, HMGCL, GYS1, TPR, ACP5, ARSB, SULT2A1, BCHE, MECR, RRM2, CKMT1A, ACOT13, FABP1, GRHPR, FABP2, RBP2, GSTA3, FABP6, GSTA1, GLA, GAPDH, LGMN | 2.2393 | 1.91E-03 |
| GOTERM_BP_DIRECT | GO:0070098~chemokine-mediated signaling pathway | 9 | 1.26E-05 | CCL25, CXCL11, CXCL9, CCL8, CCL11, CXCL8, CCL7, CCL4, CCL2 | 7.6522 | 3.87E-03 |
| GOTERM_BP_DIRECT | GO:0030593~neutrophil chemotaxis | 10 | 1.53E-05 | CCL25, LGALS3, CXCL11, CXCL9, CCL8, CCL11, CXCL8, CCL7, CCL4, CCL2 | 6.3768 | 3.87E-03 |
| GOTERM_BP_DIRECT | GO:0048245~eosinophil chemotaxis | 7 | 3.54E-05 | CCL25, LGALS3, CCL8, CCL11, CCL7, CCL4, CCL2 | 10.2029 | 6.71E-03 |
| GOTERM_MF_DIRECT | GO:0008009~chemokine activity | 9 | 3.76E-05 | CCL25, CXCL11, CXCL9, CCL8, CCL11, CXCL8, CCL7, CCL4, CCL2 | 6.6508 | 8.69E-03 |
| REACTOME_PATHWAY | R-HSA-380108~Chemokine receptors bind chemokines | 8 | 4.47E-05 | CCL25, CXCL11, CXCL9, CCL11, CXCL8, CCL7, CCL4, CCL2 | 7.6989 | 8.93E-03 |
| KEGG_PATHWAY | hsa01100:Metabolic pathways | 25 | 1.00E-04 | DDC, ECHS1, MVK, ACY1, IDUA, GLO1, GCNT1, PTS, KHK, NT5C, HMGCL, GYS1, ACP5, ARSB, MECR, RRM2, NOS2, CKMT1A, PMM2, GRHPR, GSTA3, GSTA1, GLA, GAPDH, NIT2 | 2.1635 | 1.73E-02 |
| WIKIPATHWAYS | WP5095:Overview of proinflammatory and profibrotic mediators | 13 | 1.43E-04 | CCL25, CXCL9, CCL11, CXCL8, AREG, VEGFA, CXCL11, CCL8, IFNL1, CCL7, CCL4, CCL2, IL17C | 3.5693 | 3.68E-02 |
| GOTERM_BP_DIRECT | GO:0071346~cellular response to type II interferon | 9 | 2.68E-04 | CCL25, CCL8, CCL11, CCL7, NOS2, CCL4, CCL2, LGALS9, GAPDH | 5.1014 | 3.85E-02 |
| GOTERM_BP_DIRECT | GO:0006935~chemotaxis | 9 | 3.05E-04 | CCL25, CXCL11, CXCL9, CCL8, CCL11, CXCL8, CCL7, CCL2, LGALS9 | 5.0087 | 3.85E-02 |

1. **Pathway Enrichment Analysis - Transcriptomics**

The following table lists the significant enriched pathways identified from the transcriptomics data, where enrichment analysis was performed on the top 100 most significant genes, with only those pathways having an FDR-corrected p-value of less than 0.05 and downregulated in the DTG + 3TC group compared to the 3DR group were included.

**Table S12.** Significant pathways (FDR < 0.05) for the genes in DTG + 3TC compared to DTG + 3TC + ABC

| **Term ID** | **Description** | **P-value** | **FDR** | **q-vlue** | **Genes** | **Count** |
| --- | --- | --- | --- | --- | --- | --- |
| HALLMARK_TNFA_SIGNALING_VIA_NFKB | HALLMARK_TNFA_SIGNALING_VIA_NFKB | 1.08E-15 | 3.88E-14 | 3.40E-14 | 3553/388/3725/1958/3164/467/7124/6446/969/7538/2354/1959/10957/9592/1880/10769/7422/7262/4616/3726 | 20 |
| hsa05417 | Lipid and atherosclerosis | 3.78E-09 | 6.00E-07 | 3.74E-07 | 3553/3304/3725/7124/3312/54205/1649/6348/3320/3329/3576 | 11 |
| hsa05134 | Legionellosis | 2.39E-08 | 3.79E-06 | 1.18E-06 | 3553/3304/7124/3312/54205/3329/3576 | 7 |
| hsa04657 | IL-17 signaling pathway | 9.90E-08 | 1.57E-05 | 3.26E-06 | 3553/3725/7124/3458/2354/3320/3576 | 7 |
| hsa05323 | Rheumatoid arthritis | 2.13E-07 | 3.38E-05 | 5.26E-06 | 3553/3725/7124/3458/7422/6348/3576 | 7 |
| GO:0044183 | protein folding chaperone | 4.90E-07 | 9.60E-05 | 7.00E-05 | HSPA1B/DNAJB1/HSPA8/HSP90AA1/HSPE1/HSPD1 | 6 |
| GO:0043618 | regulation of transcription from RNA polymerase II promoter in response to stress | 5.13E-08 | 1.21E-04 | 9.00E-05 | JUN/EGR1/ATF3/DDIT3/VEGFA/CITED2 | 6 |
| GO:0140662 | ATP-dependent protein folding chaperone | 8.39E-07 | 1.64E-04 | 7.00E-05 | HSPA1B/HSPA8/HSP90AA1/HSPE1/HSPD1 | 5 |
| GO:0051082 | unfolded protein binding | 1.24E-06 | 2.43E-04 | 7.00E-05 | HSPA1B/DNAJB1/HSPA8/DNAJA3/HSP90AA1/HSPE1/HSPD1 | 7 |
| GO:0043620 | regulation of DNA-templated transcription in response to stress | 1.19E-07 | 2.80E-04 | 1.04E-04 | JUN/EGR1/ATF3/DDIT3/VEGFA/CITED2 | 6 |
| hsa04612 | Antigen processing and presentation | 3.97E-06 | 6.31E-04 | 7.85E-05 | 3304/7124/10437/3458/3312/3320 | 6 |
| GO:0005125 | cytokine activity | 5.50E-06 | 1.08E-03 | 2.33E-04 | IL1B/TNF/IFNG/VEGFA/CCL3/CXCL8 | 6 |
| GO:0006986 | response to unfolded protein | 4.60E-07 | 1.09E-03 | 2.35E-04 | HSPA1B/ATF3/DNAJB1/HSPA8/DDIT3/HSP90AA1/HSPE1/HSPD1 | 8 |
| hsa04010 | MAPK signaling pathway | 7.45E-06 | 1.18E-03 | 1.11E-04 | 3553/3304/3725/3164/7124/3312/1649/7422/4616 | 9 |
| GO:0002521 | leukocyte differentiation | 5.36E-07 | 1.27E-03 | 2.35E-04 | IL1B/JUN/EGR1/TNF/IFNG/GPR18/LY9/GPR183/VEGFA/CCL3/CITED2/DNAJA3/JUNB/PHF14 | 14 |
| hsa04933 | AGE-RAGE signaling pathway in diabetic complications | 8.33E-06 | 1.32E-03 | 1.11E-04 | 3553/3725/1958/7124/7422/3576 | 6 |
| hsa05132 | Salmonella infection | 8.98E-06 | 1.43E-03 | 1.11E-04 | 3553/388/3725/7124/1781/54205/8655/3320/3576 | 9 |
| GO:0001568 | blood vessel development | 7.83E-07 | 1.85E-03 | 2.75E-04 | IL1B/RHOB/JUN/EGR1/NR4A1/TNF/EGR2/DDIT3/PLK2/VEGFA/CITED2/JUNB/CXCL8 | 13 |
| hsa05142 | Chagas disease | 1.30E-05 | 2.06E-03 | 1.43E-04 | 3553/3725/7124/3458/6348/3576 | 6 |
| GO:0005126 | cytokine receptor binding | 1.27E-05 | 2.48E-03 | 4.29E-04 | IL1B/TNF/PIBF1/IFNG/VEGFA/CCL3/CXCL8 | 7 |
| GO:0001944 | vasculature development | 1.37E-06 | 3.23E-03 | 3.70E-04 | IL1B/RHOB/JUN/EGR1/NR4A1/TNF/EGR2/DDIT3/PLK2/VEGFA/CITED2/JUNB/CXCL8 | 13 |
| GO:0010942 | positive regulation of cell death | 1.48E-06 | 3.49E-03 | 3.70E-04 | RHOB/JUN/EGR1/NR4A1/ATF3/TNF/IFNG/ID3/DDIT3/CCL3/PHLDA2/GADD45B/HSPD1 | 13 |
| GO:0035966 | response to topologically incorrect protein | 1.69E-06 | 4.00E-03 | 3.71E-04 | HSPA1B/ATF3/DNAJB1/HSPA8/DDIT3/HSP90AA1/HSPE1/HSPD1 | 8 |
| GO:0043065 | positive regulation of apoptotic process | 2.19E-06 | 5.18E-03 | 4.28E-04 | RHOB/JUN/NR4A1/ATF3/TNF/IFNG/ID3/DDIT3/CCL3/PHLDA2/GADD45B/HSPD1 | 12 |
| hsa05418 | Fluid shear stress and atherosclerosis | 3.83E-05 | 6.09E-03 | 3.79E-04 | 3553/3725/7124/3458/7422/3320 | 6 |
| GO:0035914 | skeletal muscle cell differentiation | 2.82E-06 | 6.67E-03 | 4.71E-04 | EGR1/NR4A1/ATF3/EGR2/CITED2 | 5 |
| GO:0043068 | positive regulation of programmed cell death | 3.03E-06 | 7.15E-03 | 4.71E-04 | RHOB/JUN/NR4A1/ATF3/TNF/IFNG/ID3/DDIT3/CCL3/PHLDA2/GADD45B/HSPD1 | 12 |
| GO:0006458 | 'de novo' protein folding | 3.22E-06 | 7.61E-03 | 4.71E-04 | HSPA1B/DNAJB1/HSPA8/HSPE1/HSPD1 | 5 |
| HALLMARK_APOPTOSIS | HALLMARK_APOPTOSIS | 2.26E-04 | 8.13E-03 | 3.04E-03 | 3553/388/3725/467/7124/969/1649/4616 | 8 |
| GO:0061900 | glial cell activation | 3.67E-06 | 8.66E-03 | 4.95E-04 | IL1B/JUN/TNF/IFNG/CCL3 | 5 |
| hsa04940 | Type I diabetes mellitus | 5.69E-05 | 9.04E-03 | 5.11E-04 | 3553/7124/3458/3329 | 4 |
| hsa05144 | Malaria | 6.41E-05 | 1.02E-02 | 5.29E-04 | 3553/7124/3458/3576 | 4 |
| HALLMARK_HYPOXIA | HALLMARK_HYPOXIA | 2.89E-04 | 1.04E-02 | 3.04E-03 | 3725/467/7538/123/10957/1649/7422/10370 | 8 |
| GO:0002573 | myeloid leukocyte differentiation | 4.60E-06 | 1.09E-02 | 5.77E-04 | JUN/TNF/IFNG/GPR183/VEGFA/CCL3/CITED2/JUNB | 8 |
| GO:0042026 | protein refolding | 6.46E-06 | 1.53E-02 | 7.56E-04 | HSPA1B/HSPA8/HSP90AA1/HSPD1 | 4 |
| hsa04380 | Osteoclast differentiation | 1.13E-04 | 1.80E-02 | 8.60E-04 | 3553/3725/7124/3458/2354/3726 | 6 |
| GO:0140416 | transcription regulator inhibitor activity | 9.81E-05 | 1.92E-02 | 2.77E-03 | ID3/DNAJB1/DDIT3 | 3 |
| GO:0001819 | positive regulation of cytokine production | 8.40E-06 | 1.98E-02 | 9.21E-04 | IL1B/HSPA1B/EGR1/TNF/PIBF1/IFNG/LY9/DDIT3/CCL3/HSP90AA1/HSPD1 | 11 |
| GO:0001659 | temperature homeostasis | 9.02E-06 | 2.13E-02 | 9.32E-04 | IL1B/EGR1/TNF/ARRDC3/DDIT3/VEGFA/FTO | 7 |
| GO:0006457 | protein folding | 1.01E-05 | 2.39E-02 | 9.54E-04 | HSPA1B/DNAJB1/HSPA8/CWC27/DNAJA3/HSP90AA1/HSPE1/HSPD1 | 8 |
| GO:2000147 | positive regulation of cell motility | 1.03E-05 | 2.44E-02 | 9.54E-04 | IL1B/RHOB/JUN/TNF/IFNG/MALAT1/PTP4A1/PLK2/VEGFA/CCL3/CXCL8 | 11 |
| GO:0001228 | DNA-binding transcription activator activity, RNA polymerase II-specific | 1.26E-04 | 2.46E-02 | 2.88E-03 | JUN/EGR1/NR4A1/ATF3/FOSB/EGR2/DDIT3/JUNB | 8 |
| GO:0150076 | neuroinflammatory response | 1.13E-05 | 2.66E-02 | 9.89E-04 | IL1B/JUN/TNF/IFNG/CCL3 | 5 |
| GO:0001216 | DNA-binding transcription activator activity | 1.36E-04 | 2.66E-02 | 2.88E-03 | JUN/EGR1/NR4A1/ATF3/FOSB/EGR2/DDIT3/JUNB | 8 |
| hsa05164 | Influenza A | 1.68E-04 | 2.68E-02 | 1.13E-03 | 3553/7124/3458/3337/54205/3576 | 6 |
| hsa04620 | Toll-like receptor signaling pathway | 1.77E-04 | 2.81E-02 | 1.13E-03 | 3553/3725/7124/6348/3576 | 5 |
| GO:0040017 | positive regulation of locomotion | 1.20E-05 | 2.84E-02 | 1.01E-03 | IL1B/RHOB/JUN/TNF/IFNG/MALAT1/PTP4A1/PLK2/VEGFA/CCL3/CXCL8 | 11 |
| hsa04932 | Non-alcoholic fatty liver disease | 1.83E-04 | 2.92E-02 | 1.13E-03 | 3553/3725/7124/54205/1649/3576 | 6 |
| GO:0030099 | myeloid cell differentiation | 1.27E-05 | 3.00E-02 | 1.01E-03 | HSPA1B/JUN/TNF/IFNG/ZFP36/GPR183/VEGFA/CCL3/CITED2/JUNB | 10 |
| GO:1903131 | mononuclear cell differentiation | 1.33E-05 | 3.14E-02 | 1.01E-03 | IL1B/JUN/EGR1/IFNG/GPR18/LY9/GPR183/VEGFA/DNAJA3/JUNB/PHF14 | 11 |
| GO:0048018 | receptor ligand activity | 2.06E-04 | 4.03E-02 | 3.87E-03 | IL1B/TNF/IFNG/VEGFA/CCL3/CXCL8 | 6 |
| hsa05020 | Prion disease | 2.77E-04 | 4.41E-02 | 1.62E-03 | 3553/3304/1958/7124/3312/54205/1649 | 7 |
| hsa05321 | Inflammatory bowel disease | 2.95E-04 | 4.70E-02 | 1.62E-03 | 3553/3725/7124/3458 | 4 |
| GO:0030546 | signaling receptor activator activity | 2.46E-04 | 4.83E-02 | 4.17E-03 | IL1B/TNF/IFNG/VEGFA/CCL3/CXCL8 | 6 |

**Table S13.** Significant pathways (FDR < 0.05) for the genes in DTG + 3TC compared to DTG + TDF/TAF + FTC

| **Term ID** | **Description** | **P-value** | **FDR** | **q-vlue** | **Genes** | **Count** |
| --- | --- | --- | --- | --- | --- | --- |
| GO:0001774 | microglial cell activation | 1.62E-05 | 2.63E-02 | 9.62E-03 | JUN/CX3CR1/IFNG/CCL3 | 4 |
| GO:0010942 | positive regulation of cell death | 1.75E-05 | 2.84E-02 | 9.62E-03 | RHOB/EGR1/JUN/TXNIP/G0S2/GADD45B/DFFA/UCP2/IFNG/CCL3 | 10 |
| GO:0002269 | leukocyte activation involved in inflammatory response | 2.03E-05 | 3.30E-02 | 9.62E-03 | JUN/CX3CR1/IFNG/CCL3 | 4 |
| GO:0061900 | glial cell activation | 2.78E-05 | 4.52E-02 | 9.88E-03 | JUN/CX3CR1/IFNG/CCL3 | 4 |
| GO:0042379 | chemokine receptor binding | 1.20E-04 | 2.17E-02 | 1.91E-02 | CXCL8/CX3CR1/CCL3 | 3 |
| hsa05323 | Rheumatoid arthritis | 2.89E-05 | 4.37E-03 | 3.62E-03 | 3725/51382/3576/3458/6348 | 5 |
| hsa04061 | Viral protein interaction with cytokine and cytokine receptor | 2.39E-04 | 3.61E-02 | 1.50E-02 | 3576/1524/6348/3561 | 4 |
| HALLMARK_TNFA_SIGNALING_VIA_NFKB | HALLMARK_TNFA_SIGNALING_VIA_NFKB | 8.75E-05 | 3.32E-03 | 3.13E-03 | 388/1958/3725/7071/50486/7538/4616/6446 | 8 |

**Table S14.** Significant pathways (FDR < 0.05) for the genes in DTG + 3TC compared to DTG + TDF + FTC

| **Term ID** | **Description** | **P-value** | **FDR** | **q-vlue** | **Genes** | **Count** |
| --- | --- | --- | --- | --- | --- | --- |
| GO:0042571 | immunoglobulin complex, circulating | 2.26E-08 | 3.24E-06 | 3.14E-06 | IGHV4-34/IGHG1/TRDC/IGHV5-51/IGHG3/IGHD | 6 |
| GO:0034987 | immunoglobulin receptor binding | 3.73E-08 | 6.49E-06 | 6.25E-06 | IGHV4-34/IGHG1/TRDC/IGHV5-51/IGHG3/IGHD | 6 |
| GO:0019814 | immunoglobulin complex | 1.45E-06 | 2.08E-04 | 1.01E-04 | IGHV4-34/IGHG1/TRDC/IGHV5-51/IGHG3/IGHD | 6 |
| GO:0006910 | phagocytosis, recognition | 1.85E-07 | 2.22E-04 | 1.30E-04 | IGHV4-34/IGHG1/TRDC/IGHV5-51/IGHG3/IGHD | 6 |
| GO:0006958 | complement activation, classical pathway | 3.02E-07 | 3.62E-04 | 1.30E-04 | IGHV4-34/IGHG1/TRDC/IGHV5-51/IGHG3/IGHD | 6 |
| GO:0050853 | B cell receptor signaling pathway | 3.34E-07 | 4.01E-04 | 1.30E-04 | IGHV4-34/IGHG1/TRDC/IGHV5-51/IGHG3/GCSAM/IGHD | 7 |
| GO:0016064 | immunoglobulin mediated immune response | 8.27E-07 | 9.92E-04 | 1.62E-04 | IGHV4-34/IGHG1/HLA-DOA/MSH2/TRDC/IGHV5-51/IGHG3/IGHD | 8 |
| GO:0002455 | humoral immune response mediated by circulating immunoglobulin | 8.76E-07 | 1.05E-03 | 1.62E-04 | IGHV4-34/IGHG1/TRDC/IGHV5-51/IGHG3/IGHD | 6 |
| GO:0006956 | complement activation | 8.76E-07 | 1.05E-03 | 1.62E-04 | IGHV4-34/IGHG1/TRDC/IGHV5-51/IGHG3/IGHD | 6 |
| GO:0019724 | B cell mediated immunity | 9.69E-07 | 1.16E-03 | 1.62E-04 | IGHV4-34/IGHG1/HLA-DOA/MSH2/TRDC/IGHV5-51/IGHG3/IGHD | 8 |
| GO:0050871 | positive regulation of B cell activation | 1.15E-06 | 1.38E-03 | 1.68E-04 | IGHV4-34/IGHG1/MSH2/TRDC/IGHV5-51/IGHG3/IGHD | 7 |
| GO:0008037 | cell recognition | 2.56E-06 | 3.07E-03 | 3.33E-04 | IGHV4-34/IGHG1/TRDC/IGHV5-51/IGHG3/CCT3/IGHD | 7 |
| GO:0003823 | antigen binding | 1.86E-05 | 3.23E-03 | 1.55E-03 | IGHV4-34/IGHG1/TRDC/IGHV5-51/IGHG3/IGHD | 6 |
| GO:0006911 | phagocytosis, engulfment | 3.69E-06 | 4.43E-03 | 4.32E-04 | IGHV4-34/IGHG1/TRDC/IGHV5-51/IGHG3/IGHD | 6 |
| GO:0099024 | plasma membrane invagination | 6.94E-06 | 8.32E-03 | 7.38E-04 | IGHV4-34/IGHG1/TRDC/IGHV5-51/IGHG3/IGHD | 6 |
| GO:0010324 | membrane invagination | 1.08E-05 | 1.30E-02 | 1.05E-03 | IGHV4-34/IGHG1/TRDC/IGHV5-51/IGHG3/IGHD | 6 |
| GO:0050864 | regulation of B cell activation | 1.18E-05 | 1.41E-02 | 1.06E-03 | IGHV4-34/IGHG1/MSH2/TRDC/IGHV5-51/IGHG3/IGHD | 7 |

**Table S15.** Significant pathways (FDR < 0.05) for the genes in DTG + 3TC compared to DTG + TAF + FTC

| **Term ID** | **Description** | **P-value** | **FDR** | **q-vlue** | **Genes** | **Count** |
| --- | --- | --- | --- | --- | --- | --- |
| GO:0010942 | positive regulation of cell death | 9.65E-06 | 1.61E-02 | 1.21E-02 | GADD45B/RHOB/EGR1/CCL3/TXNIP/JUN/G0S2/STK3/DFFA/IFNG | 10 |
| GO:0043065 | positive regulation of apoptotic process | 2.24E-05 | 3.74E-02 | 1.21E-02 | GADD45B/RHOB/CCL3/TXNIP/JUN/G0S2/STK3/DFFA/IFNG | 9 |
| GO:0043068 | positive regulation of programmed cell death | 2.87E-05 | 4.78E-02 | 1.21E-02 | GADD45B/RHOB/CCL3/TXNIP/JUN/G0S2/STK3/DFFA/IFNG | 9 |
| GO:0035035 | histone acetyltransferase binding | 7.44E-05 | 1.44E-02 | 1.23E-02 | EGR1/CITED2/BCAS3 | 3 |
| hsa05323 | Rheumatoid arthritis | 4.01E-05 | 6.25E-03 | 5.27E-03 | 51382/3576/6348/3725/3458 | 5 |
| HALLMARK_TNFA_SIGNALING_VIA_NFKB | HALLMARK_TNFA_SIGNALING_VIA_NFKB | 7.77E-06 | 2.64E-04 | 2.53E-04 | 4616/388/1958/7538/3725/6446/50486/3726/7071 | 9 |
